# Supplementary figures and images for: Use of >100,000 NHLBI Trans-Omics for Precision Medicine (TOPMed) Consortium whole genome sequences improves imputation quality and detection of rare variant associations in admixed African and Hispanic/Latino populations
Source: PLoS Genet. 2019 Dec 23;15(12):e1008500. doi: 10.1371/journal.pgen.1008500 (PMC6953885; doi:10.1371/journal.pgen.1008500)

MAF>1%

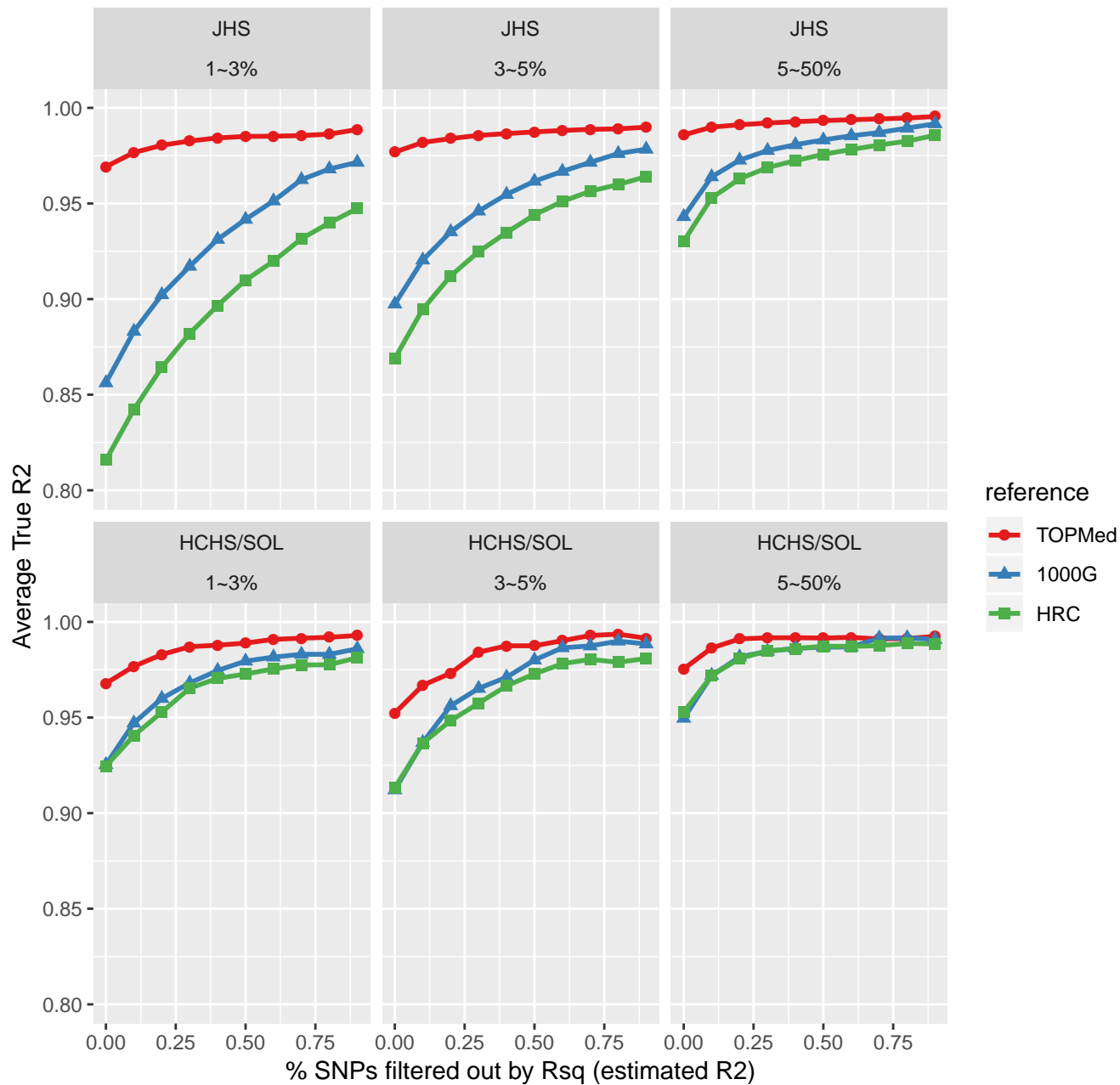

Supplement: S1 Fig — Imputation quality (measured by true R2 [Y-axis]) is plotted with progressively more stringent post-imputation filtering from left to right, with filtering according to estimated R2 (X-axis), for variants with MAF > 1%. Top panels are for the JHS cohort and bottom panels for the HCHS/SOL cohort. Three reference panels are shown: TOPMed (TOPMed freeze 5b), 1000G (the 1000 Genomes Phase 3), and HRC (the Haplotype Reference Consortium). (PDF) [file pgen.1008500.s001.pdf]

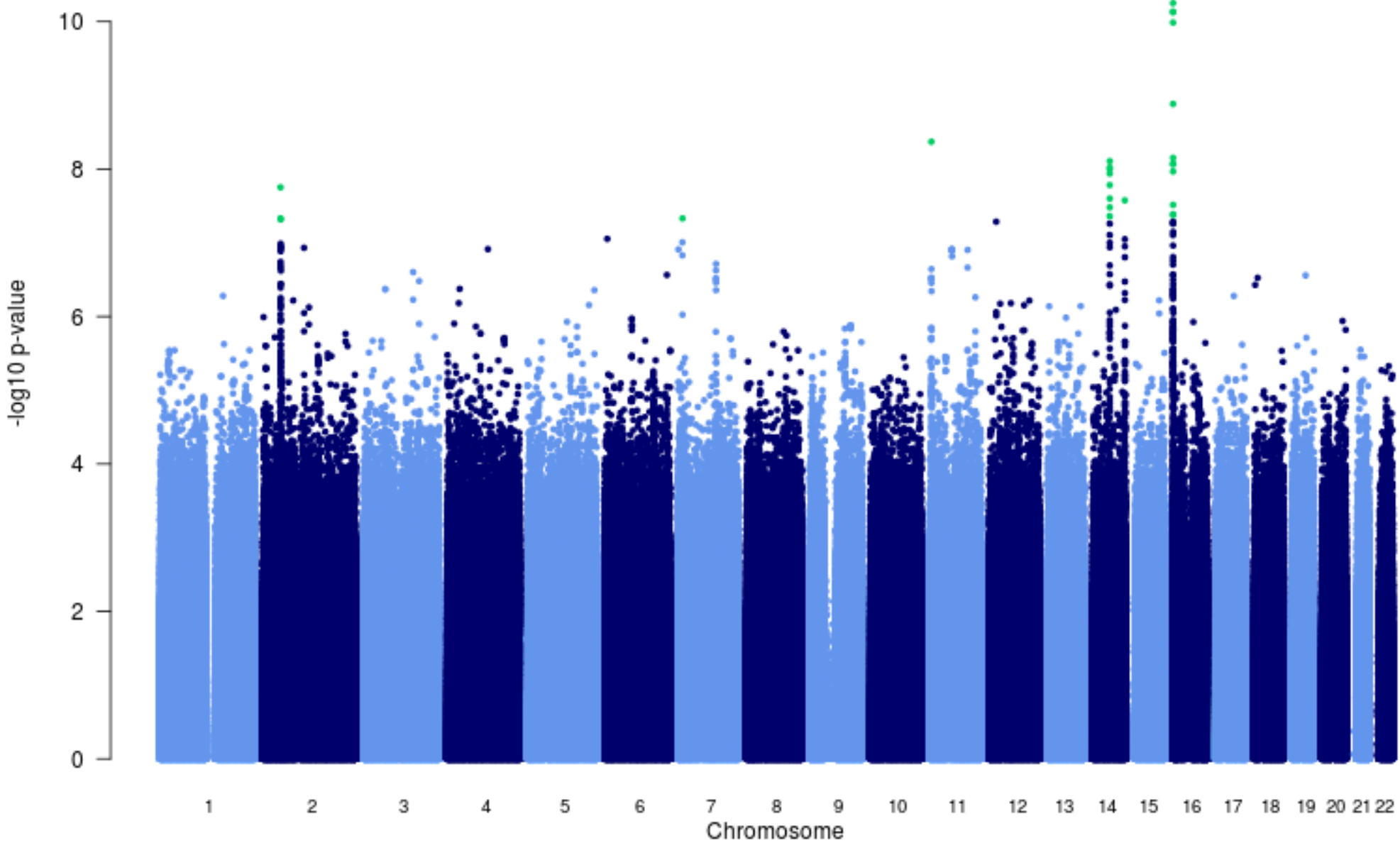

Supplement: S3 Fig — (PDF) [file pgen.1008500.s003.pdf]

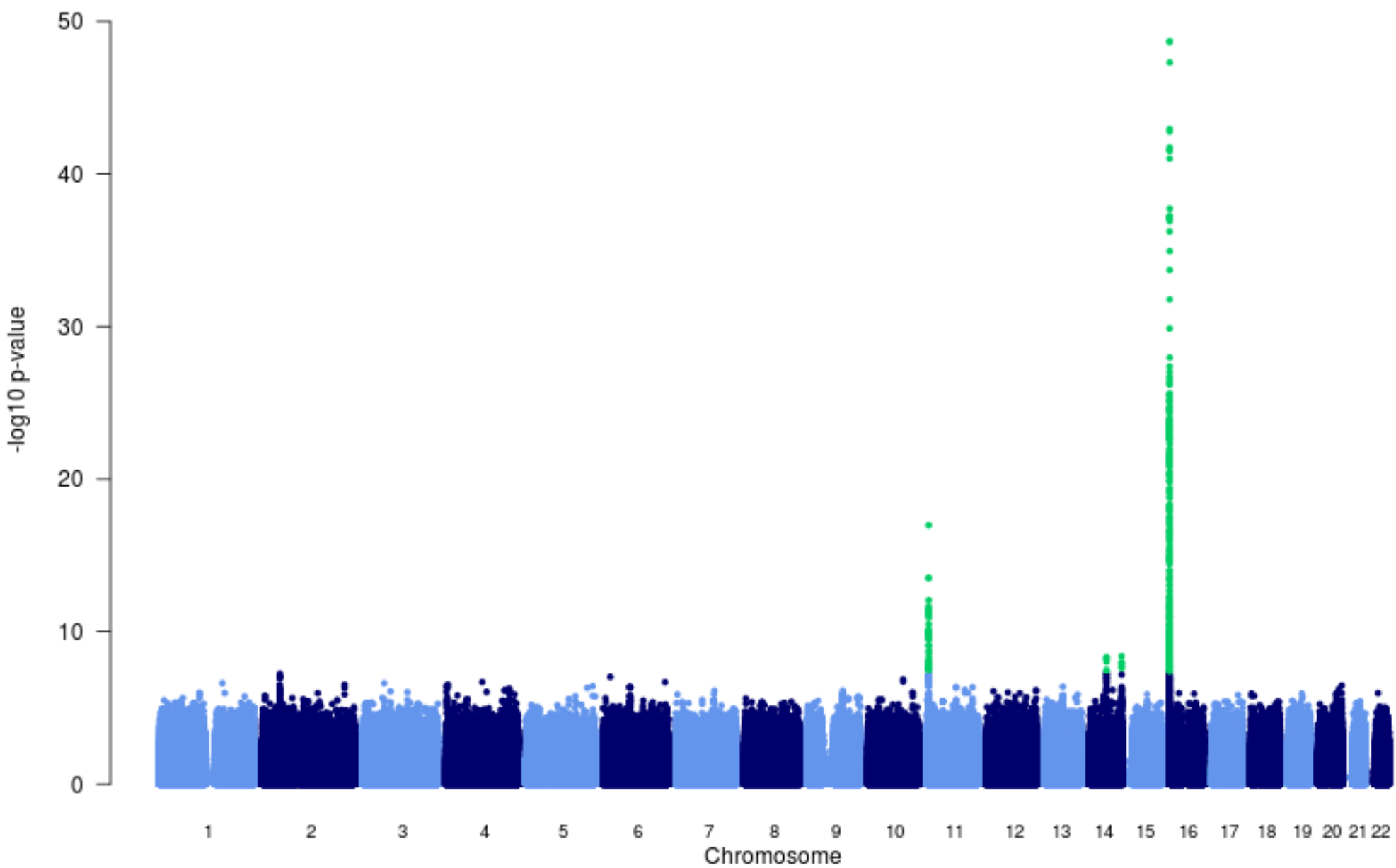

Supplement: S4 Fig — (PDF) [file pgen.1008500.s004.pdf]

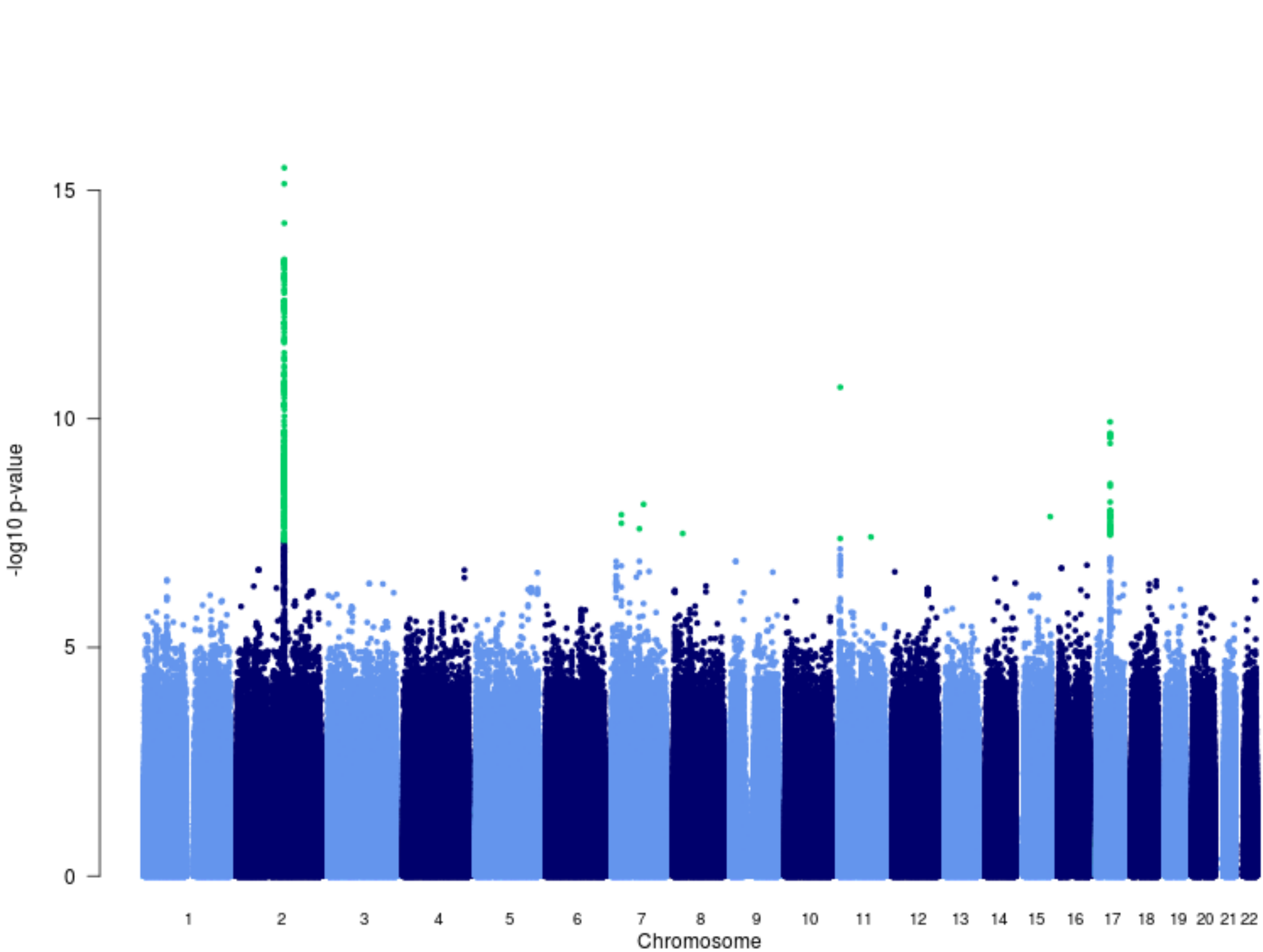

Supplement: S5 Fig — (PDF) [file pgen.1008500.s005.pdf]

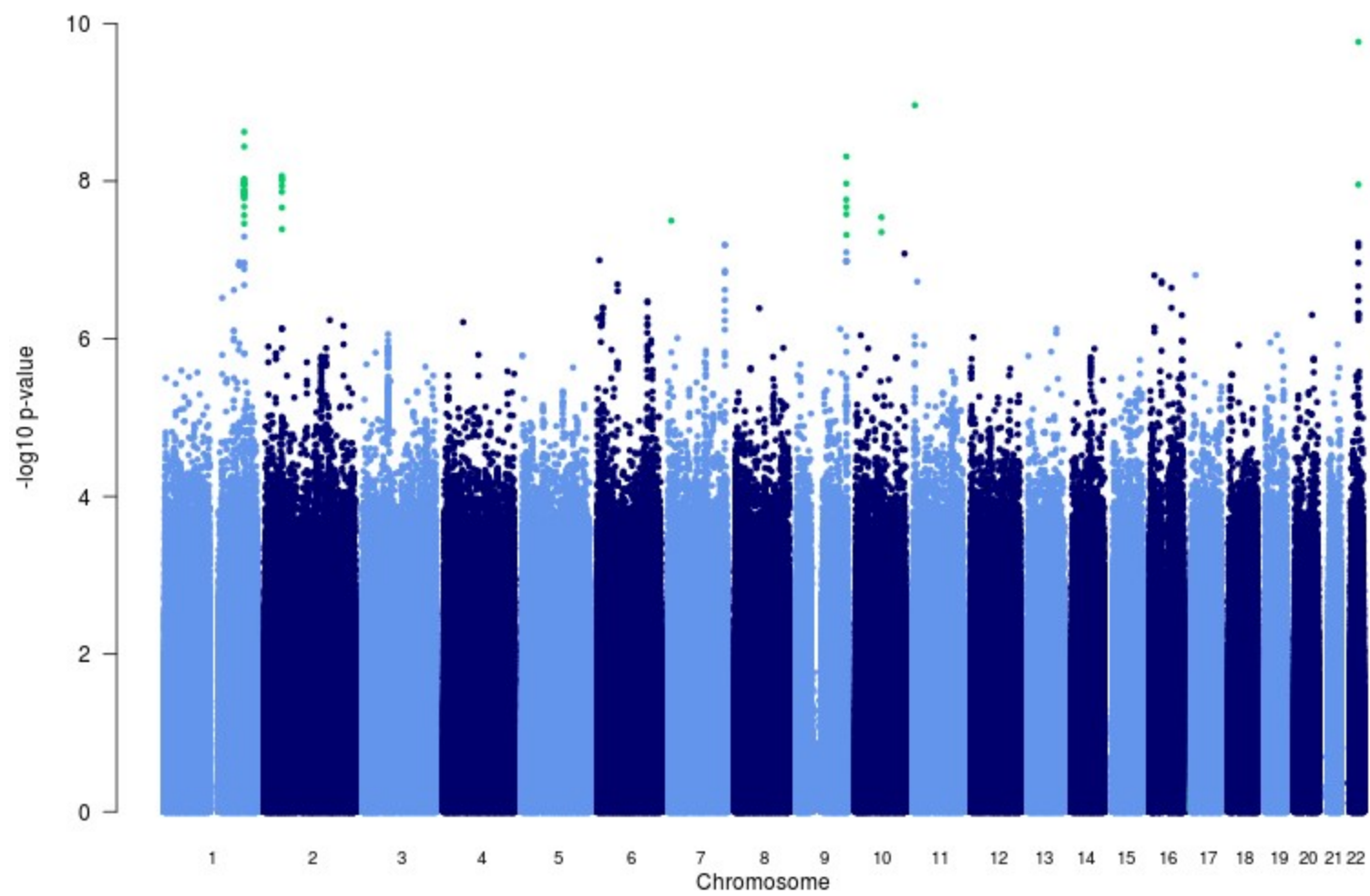

Supplement: S6 Fig — (PDF) [file pgen.1008500.s006.pdf]

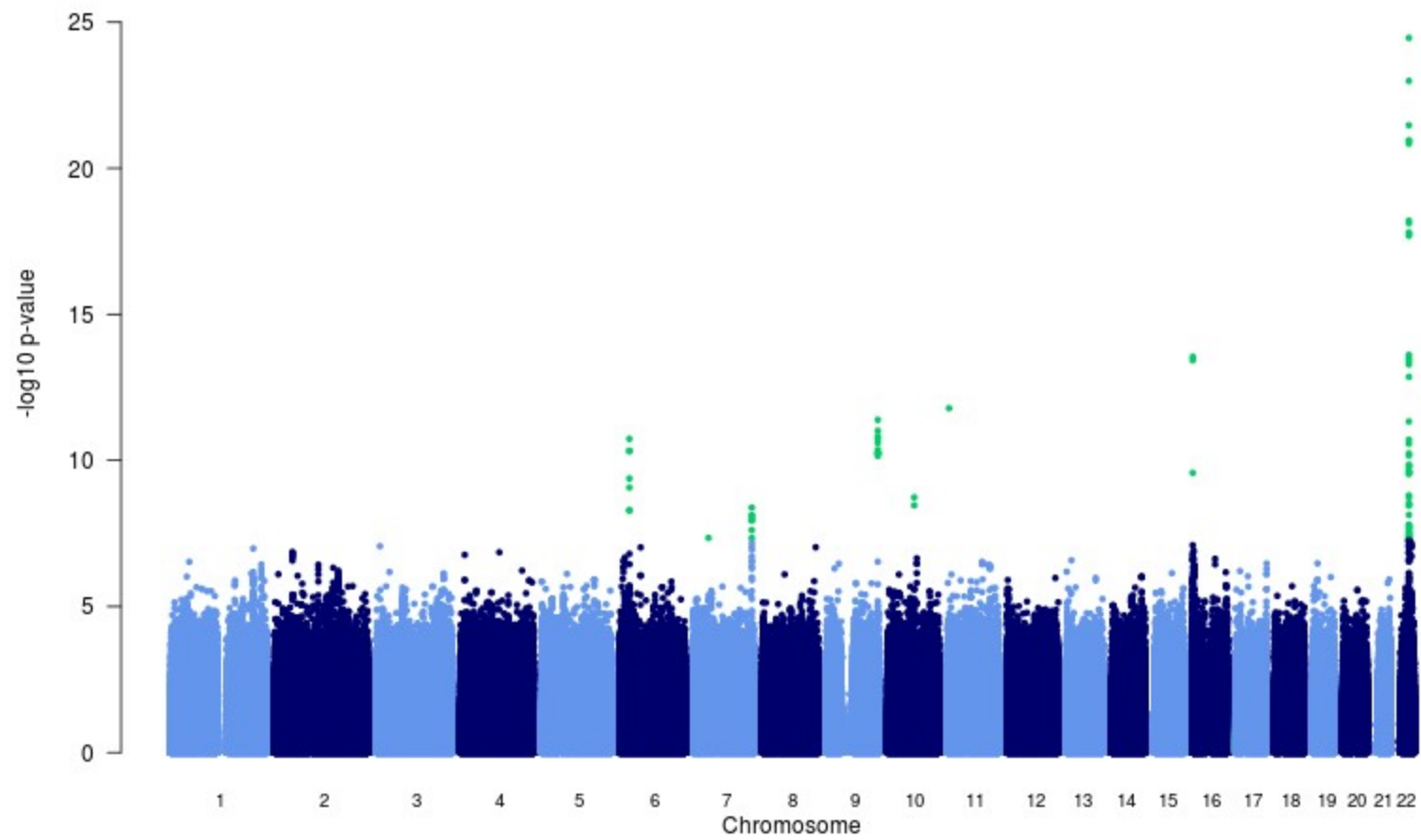

Supplement: S7 Fig — (PDF) [file pgen.1008500.s007.pdf]

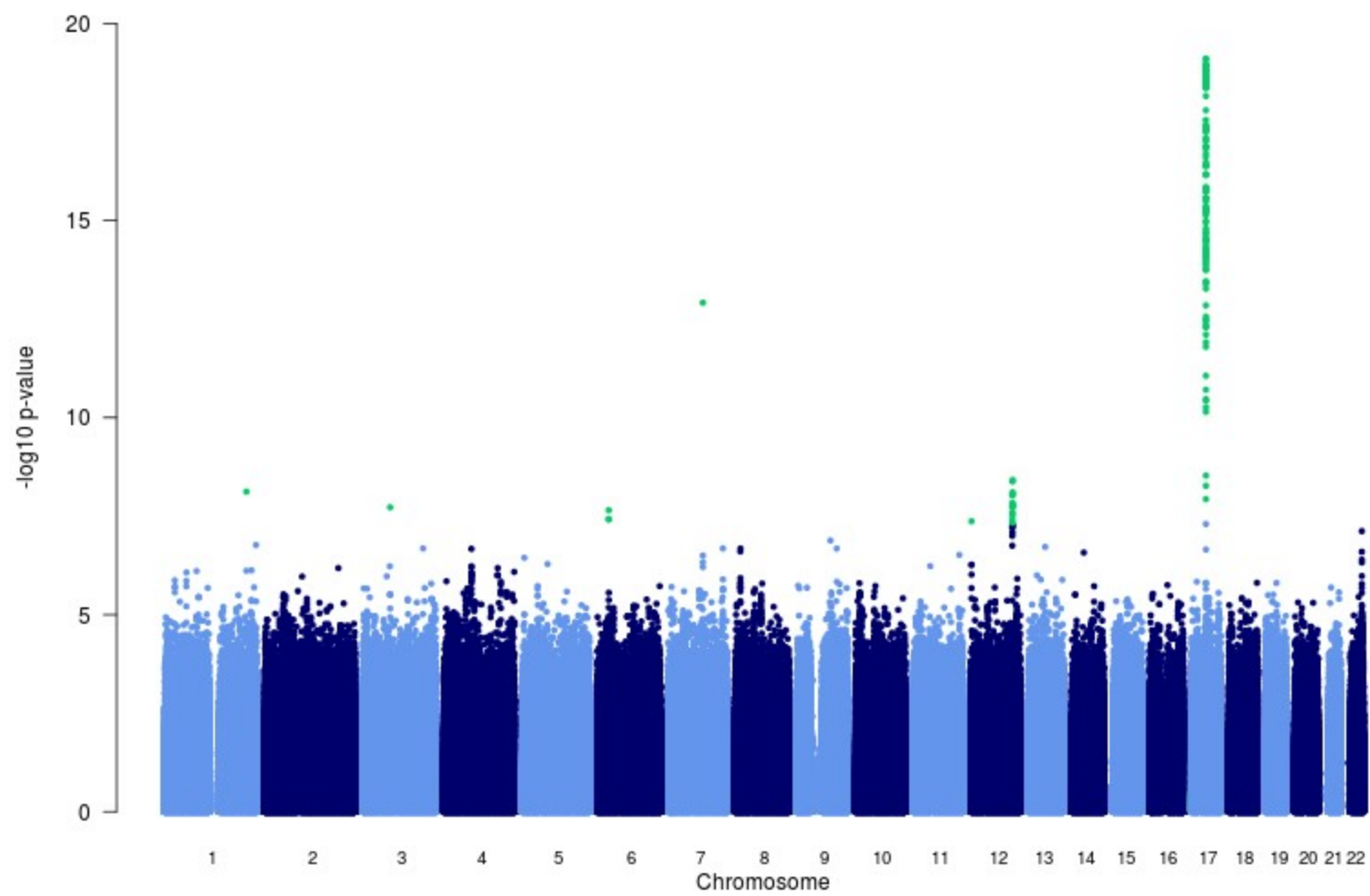

Supplement: S8 Fig — (PDF) [file pgen.1008500.s008.pdf]

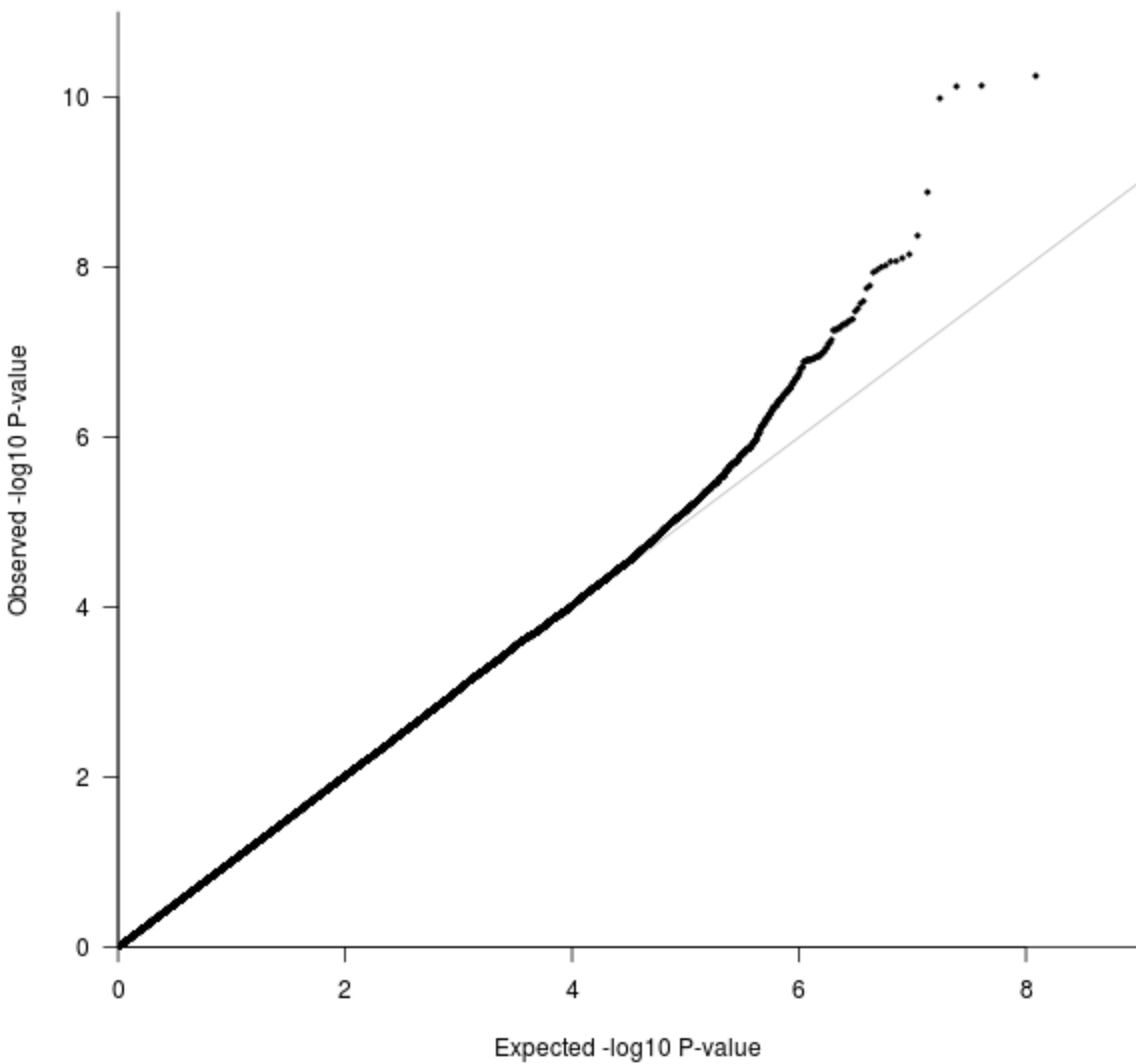

Supplement: S9 Fig — (PDF) [file pgen.1008500.s009.pdf]

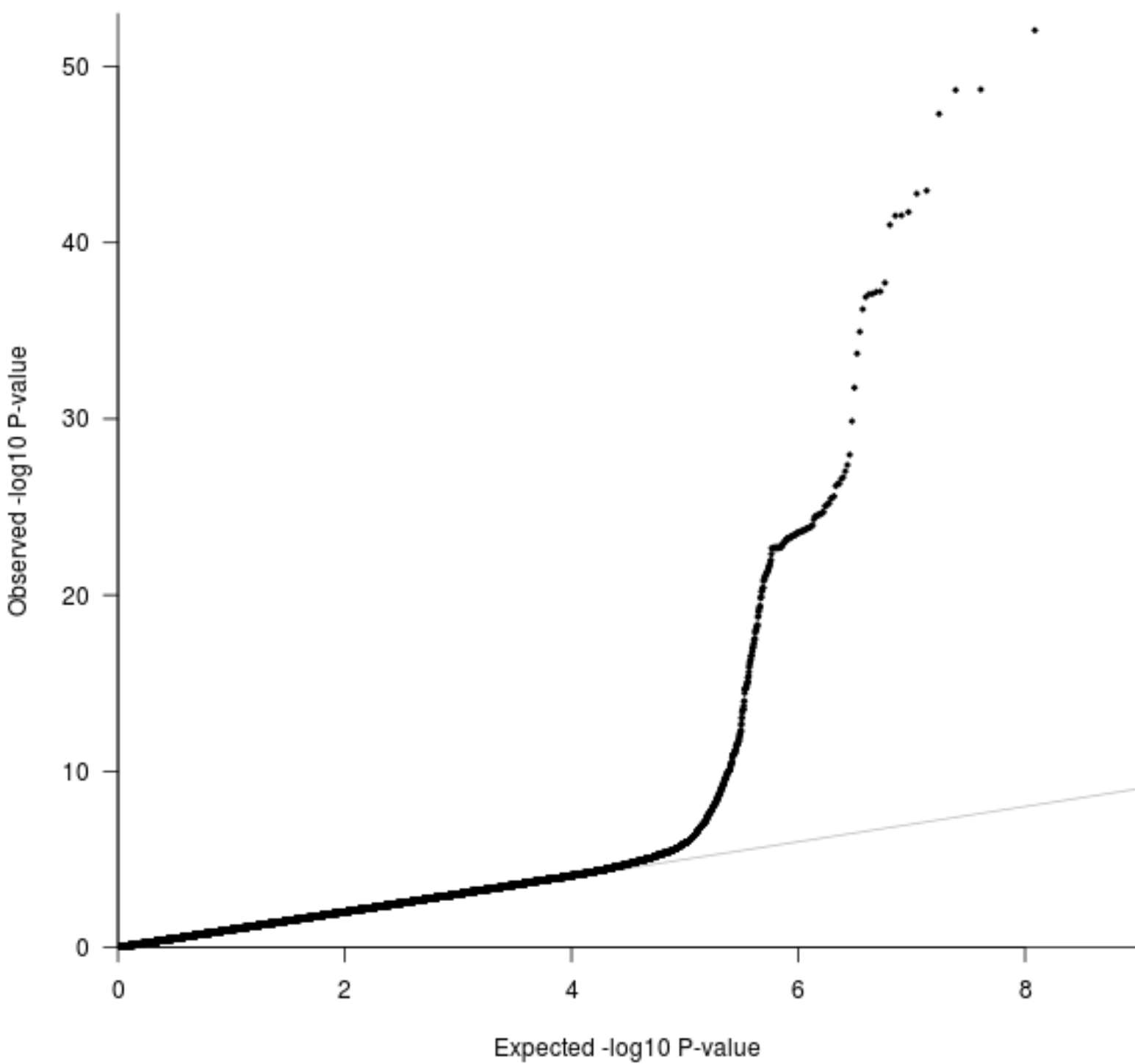

Supplement: S10 Fig — (PDF) [file pgen.1008500.s010.pdf]

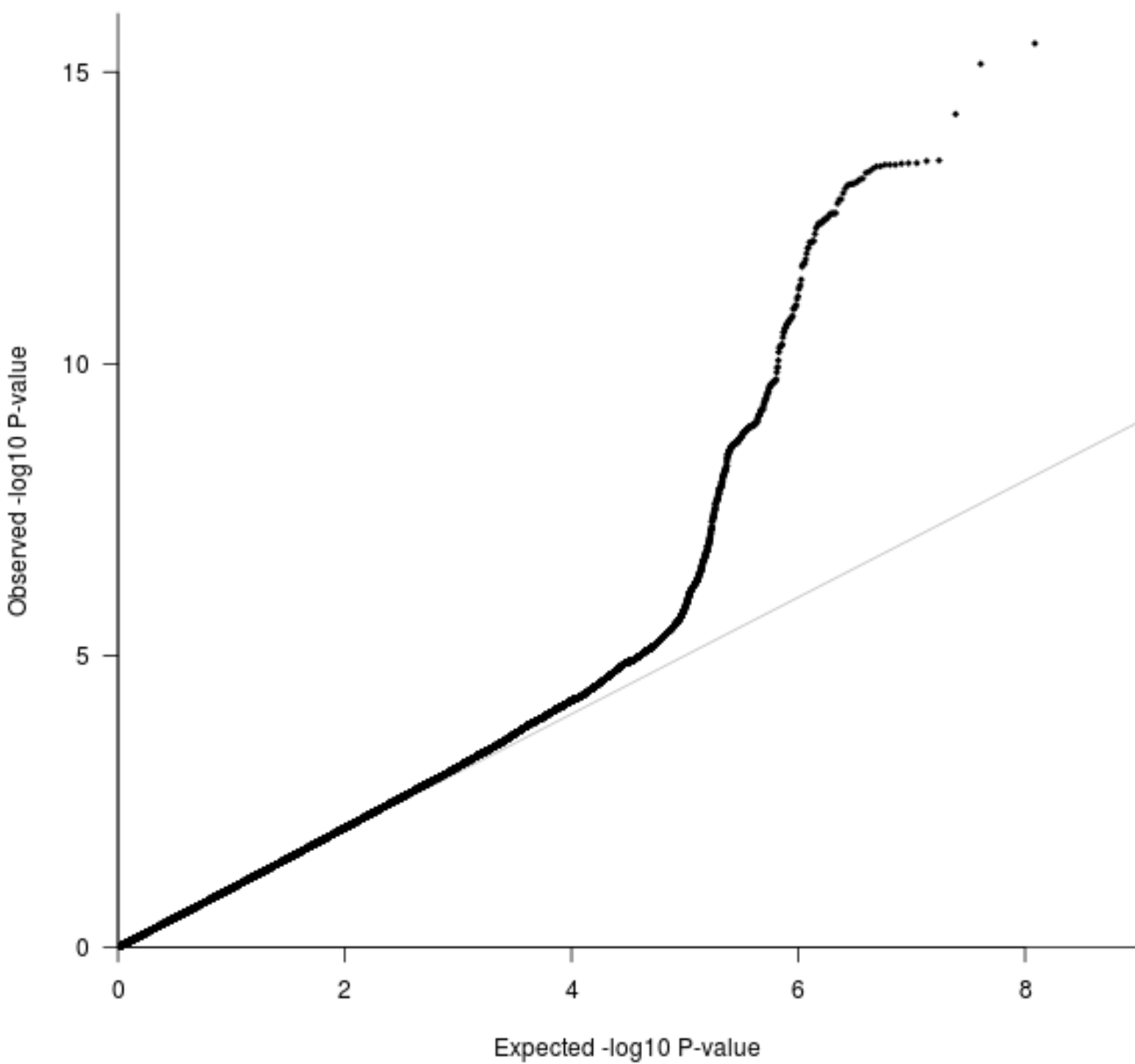

Supplement: S11 Fig — (PDF) [file pgen.1008500.s011.pdf]

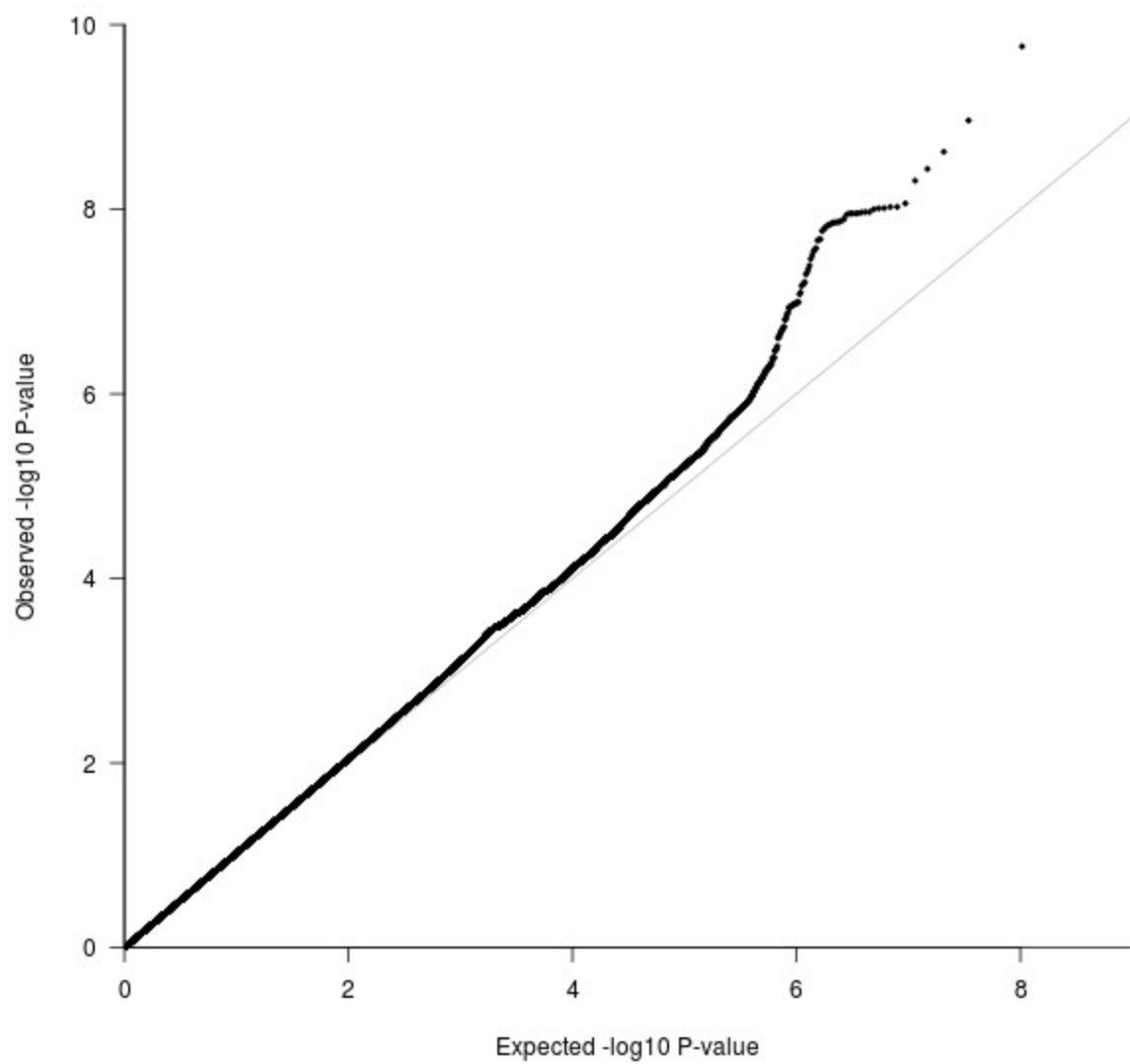

Supplement: S12 Fig — (PDF) [file pgen.1008500.s012.pdf]

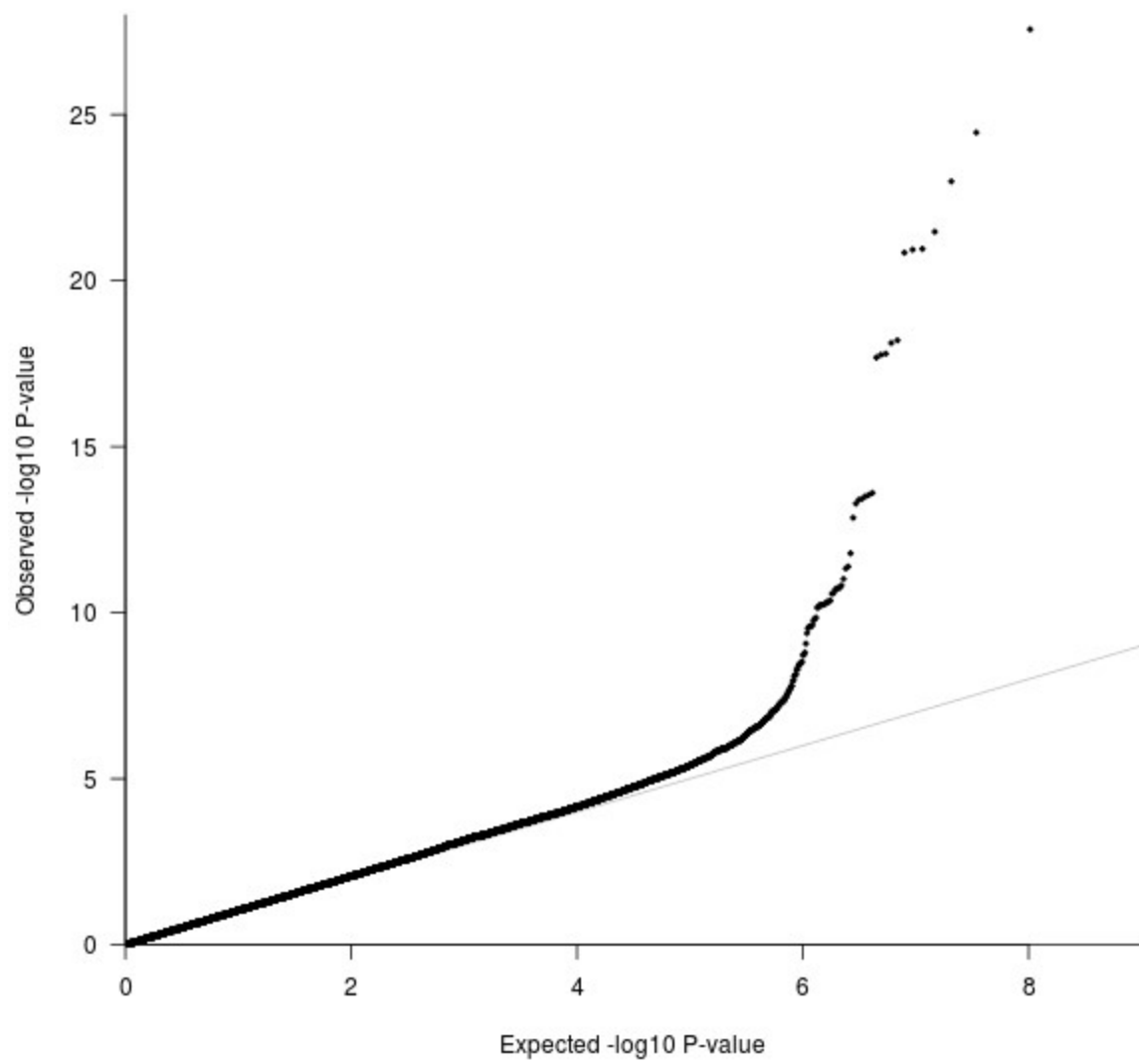

Supplement: S13 Fig — (PDF) [file pgen.1008500.s013.pdf]

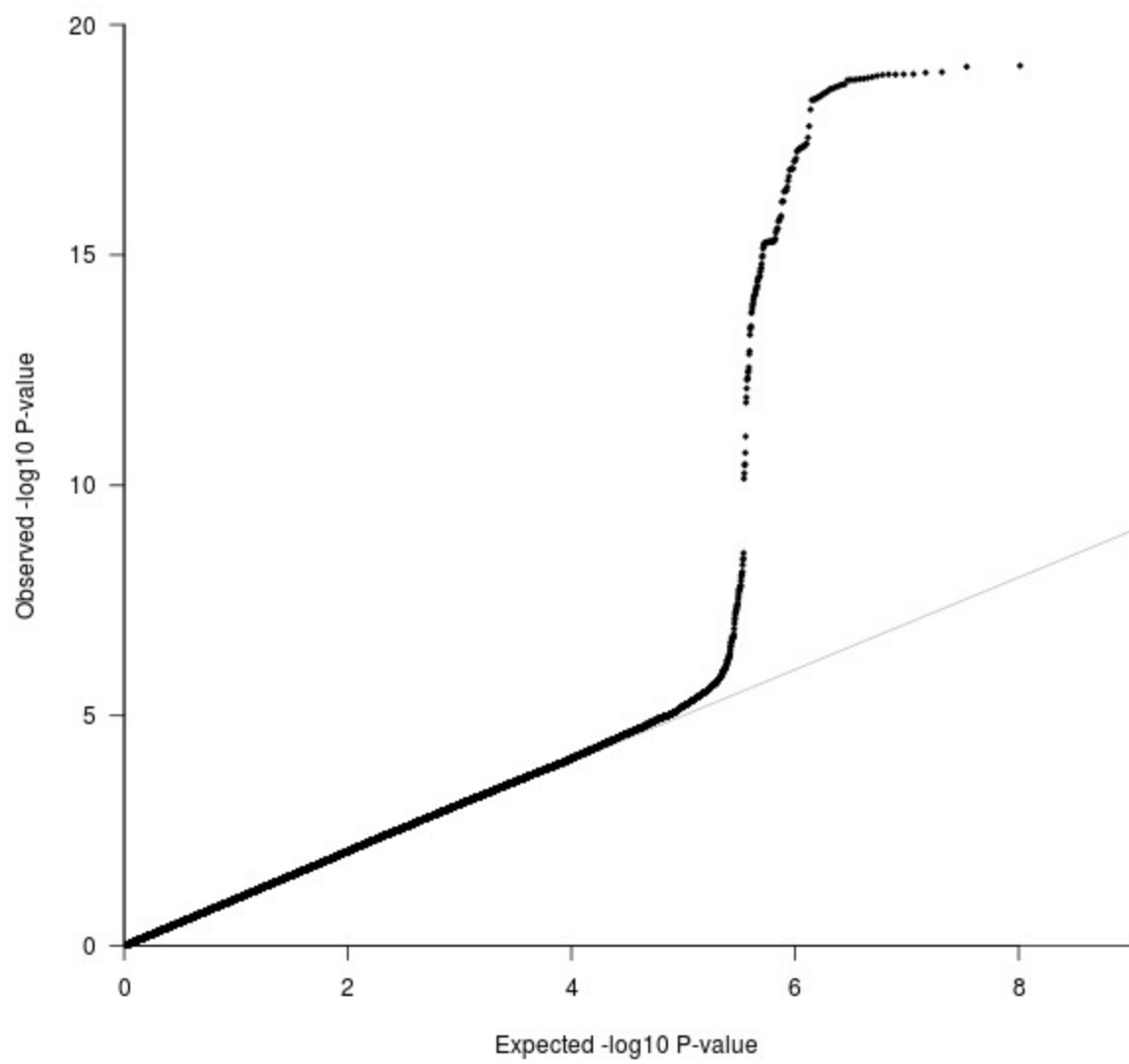

Supplement: S14 Fig — (PDF) [file pgen.1008500.s014.pdf]
