# Supplementary material for: Use of >100,000 NHLBI Trans-Omics for Precision Medicine (TOPMed) Consortium whole genome sequences improves imputation quality and detection of rare variant associations in admixed African and Hispanic/Latino populations
Source: PLoS Genet. 2019 Dec 23;15(12):e1008500. doi: 10.1371/journal.pgen.1008500 (PMC6953885; doi:10.1371/journal.pgen.1008500)

A. Comparison of all well-imputed variants included in results from TOPMed (TOPMed freeze 5b), 1000G (the 1000 Genomes Phase 3), and HRC (the Haplotype Reference Consortium) in the Jackson Heart Study (JHS).

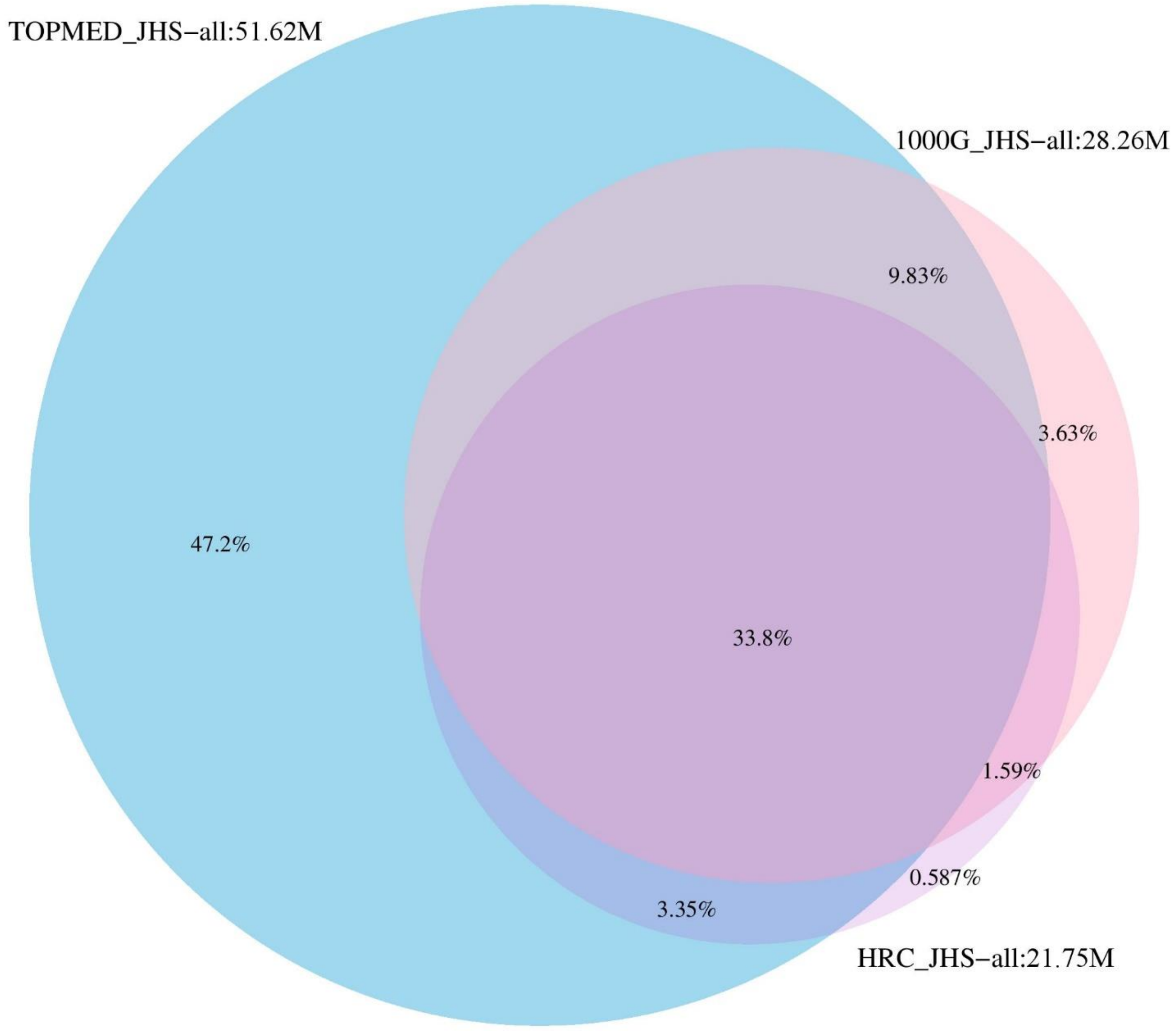

B. Comparison of all well-imputed variants with minor allele frequency < 0.5% included in results from TOPMed (TOPMed freeze 5b), 1000G (the 1000 Genomes Phase 3), and HRC (the Haplotype Reference Consortium) in the Jackson Heart Study (JHS).

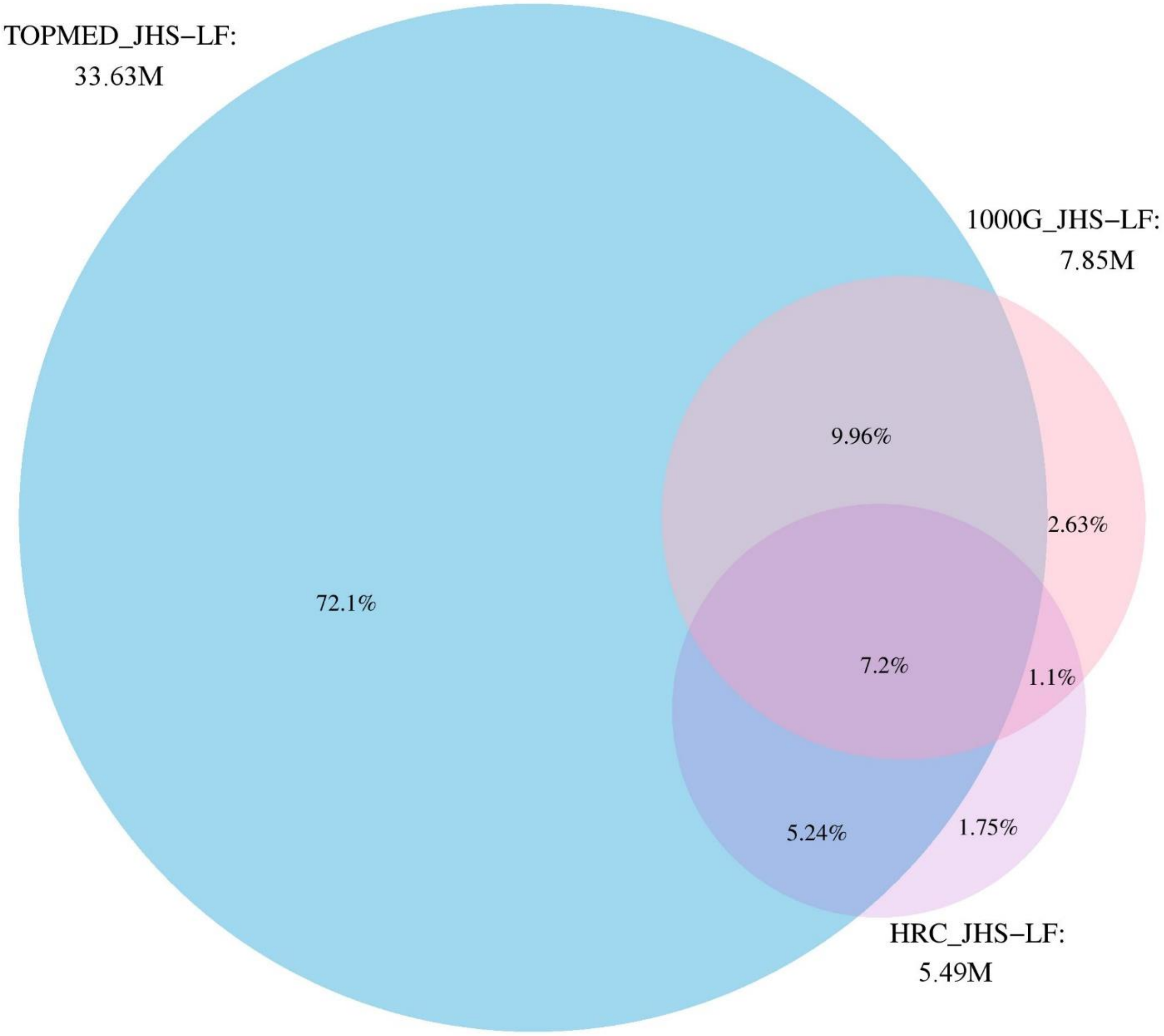

C. Comparison of all well-imputed variants with minor allele frequency < 0.05% included in results from TOPMed (TOPMed freeze 5b), 1000G (the 1000 Genomes Phase 3), and HRC (the Haplotype Reference Consortium) in the Jackson Heart Study (JHS).

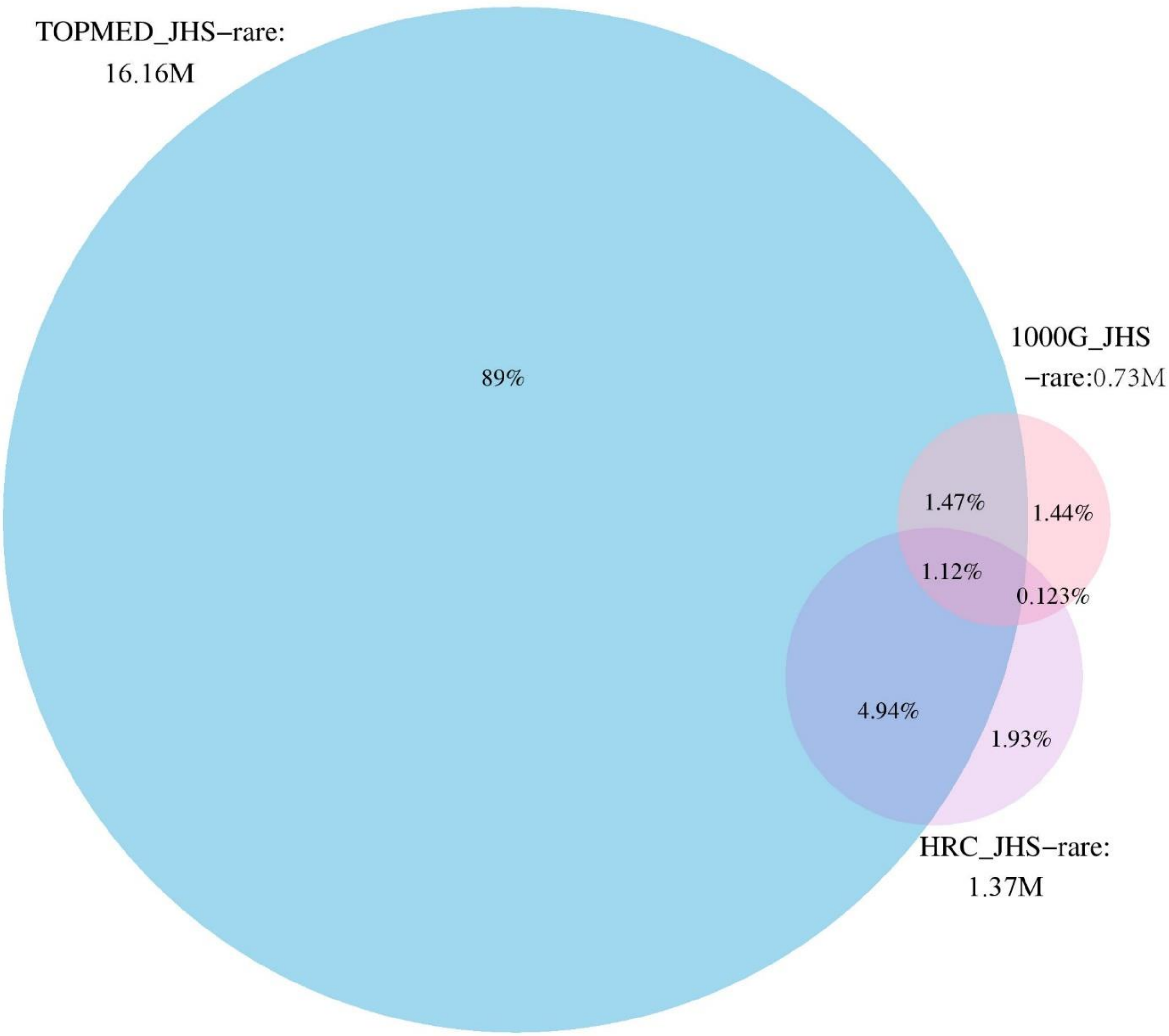

D. Comparison of all well-imputed variants included in results from TOPMed (TOPMed freeze 5b), 1000G (the 1000 Genomes Phase 3), and HRC (the Haplotype Reference Consortium) in the Hispanic Community Health Study/Study of Latinos (HCHS/SOL).

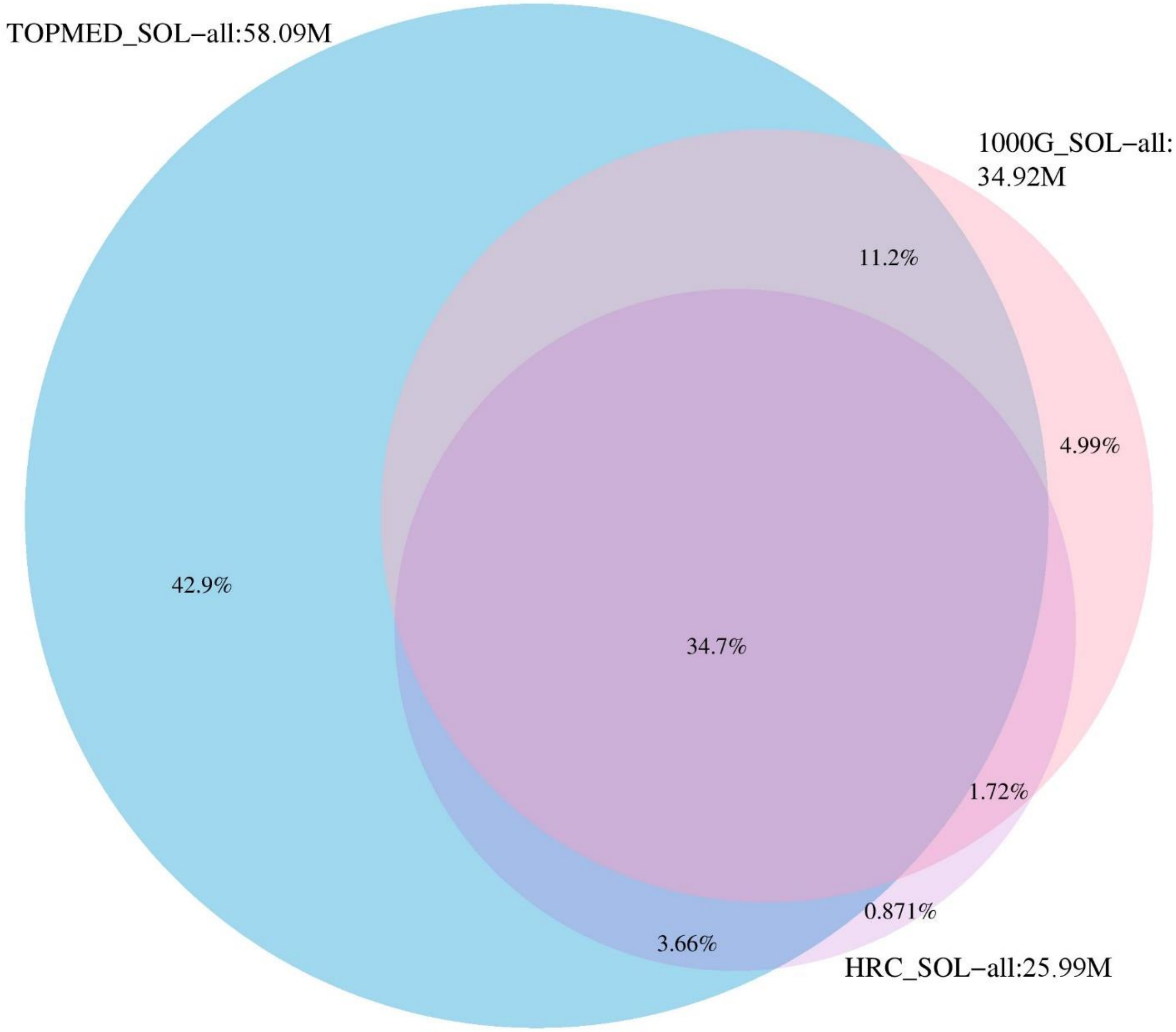

E. Comparison of all well-imputed variants with minor allele frequency < 0.5% included in results from TOPMed (TOPMed freeze 5b), 1000G (the 1000 Genomes Phase 3), and HRC (the Haplotype Reference Consortium) in the Hispanic Community Health Study/Study of Latinos (HCHS/SOL).

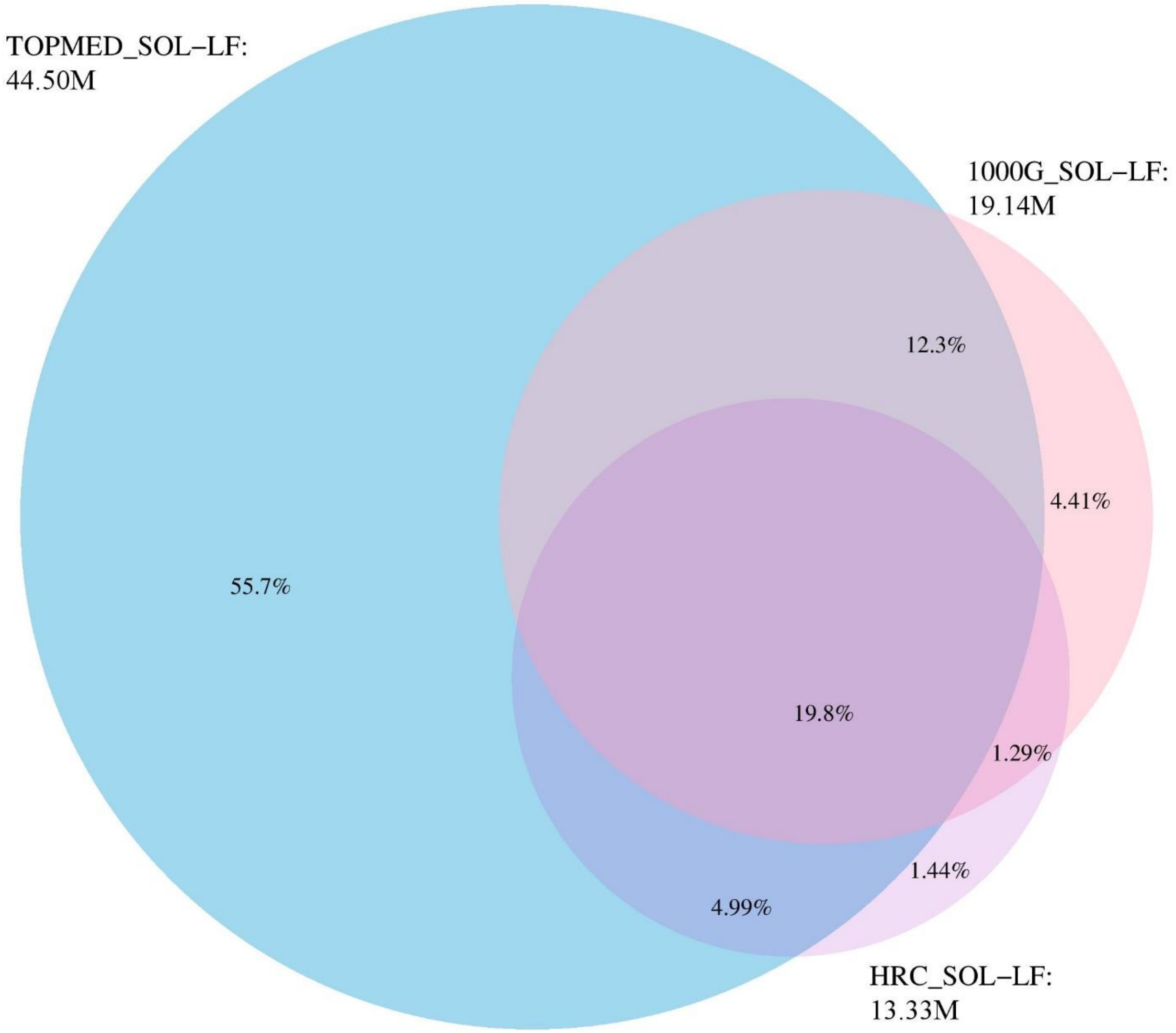

F. Comparison of all well-imputed variants with minor allele frequency < 0.05% included in results from TOPMed (TOPMed freeze 5b), 1000G (the 1000 Genomes Phase 3), and HRC (the Haplotype Reference Consortium) in the Hispanic Community Health Study/Study of Latinos (HCHS/SOL).

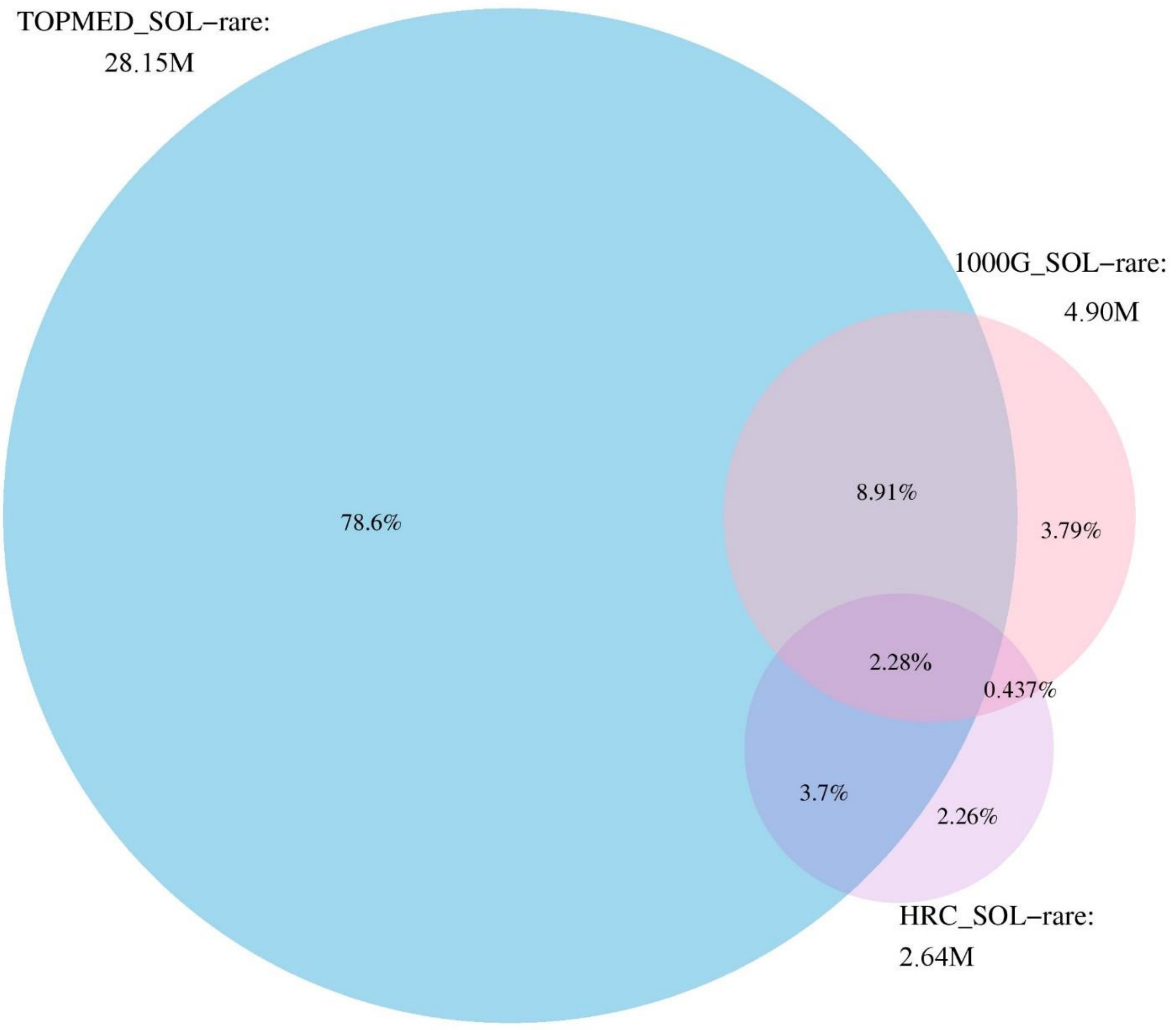

Supplement: S2 Fig — (PDF) [file pgen.1008500.s002.pdf]
