# Supplementary material for: Use of >100,000 NHLBI Trans-Omics for Precision Medicine (TOPMed) Consortium whole genome sequences improves imputation quality and detection of rare variant associations in admixed African and Hispanic/Latino populations
Source: PLoS Genet. 2019 Dec 23;15(12):e1008500. doi: 10.1371/journal.pgen.1008500 (PMC6953885; doi:10.1371/journal.pgen.1008500)
Supplement: S1 Table — (PDF) [file pgen.1008500.s015.pdf]

S1 Table. Cohorts used for imputation to TOPMed freeze 5b reference panel and subsequent association analysis with hematological traits, including self-identified African ancestry and Hispanic/Latino individuals

Cohort	Self-Identified Ancestry	Sample Size	Genotyping Array(s)	N Markers	Data Availability
WHI	African American	4,658	Affymetrix 6.0 + MEGA	2,046,432	Affymetrix 6.0 at phs000386.v7.p3, MEGA at phs000227.v5.p3
WHI	African American	2,470	Affymetrix 6.0	588,314	phs000386.v7.p3
WHI	African American	1,366	MEGA	970,993	phs000227.v5.p3
ARIC	African American	2,392	Affymetrix 6.0	782,276	phs000557.v4.p1
GERA	African American	2,246	Affymetrix Axiom (custom)	838,272	phs000674.v3.p3
UK Biobank	African	6,762	UK Biobank Axiom	733,046	By application from UK Biobank
CARDIA	African American	1619	Affymetrix 6.0	725,803	phs000613.v1.p2
WHI	Hispanic/Latino	3,237	Affymetrix 6.0 + MEGA	2,052,595	Affymetrix 6.0 at phs000386.v7.p3, MEGA at phs000227.v5.p3
WHI	Hispanic/Latino	1,081	MEGA	973,149	phs000227.v5.p3
HCHS/SOL	Hispanic/Latino	11,588	Illumina Omni2.5 + MEGA	2,144,214	phs000810.v1.p1
GERA	Hispanic/Latino	5,783	Affymetrix Axiom (custom)	752,154	phs000674.v3.p3

ARIC, Atherosclerosis Risk in Communities; CARDIA, Coronary Artery Risk Development in Young Adults; HCHS/SOL, Hispanic Community Health Study/Study of Latinos; GERA, Resource for Genetic Epidemiology Research on Aging; N markers, total number of post quality control markers used for imputation; WHI, Women's Health Initiative
