# Supplementary material for: Use of >100,000 NHLBI Trans-Omics for Precision Medicine (TOPMed) Consortium whole genome sequences improves imputation quality and detection of rare variant associations in admixed African and Hispanic/Latino populations
Source: PLoS Genet. 2019 Dec 23;15(12):e1008500. doi: 10.1371/journal.pgen.1008500 (PMC6953885; doi:10.1371/journal.pgen.1008500)
Supplement: S2 Table — (PDF) [file pgen.1008500.s016.pdf]

S2 Table. Cohorts included in the TOPMed freeze 5b imputation reference panels, with self-reported ancestry.

Study	Sample Size	dbGaP accession	Full name	African	Asian	European	Hispanic/Latino	Other	Missing
HVH	64	phs000993	Heart and Vascular Health Study (HVH)	0	0	63	0	1	0
CHS	70	phs001368	Cardiovascular Health Study	13	0	55	0	1	1
WGHS	98	phs001040	Novel Risk Factors for the Development of Atrial Fibrillation in Women	0	0	97	0	0	1
Partners	109	phs001024	Partners HealthCare Biobank	2	1	104	1	1	0
VAFAR	157	phs000997	The Vanderbilt Atrial Fibrillation Ablation Registry	0	0	154	0	0	3
DHS	324	phs001412	Diabetes Heart Study African American Coronary Artery Calcification (AA CAC)	322	0	0	0	0	2
CCAF	328	phs001189	Cleveland Clinic Atrial Fibrillation Study	0	0	325	3	0	0
Sarcoidosis	608	phs001207	African American Sarcoidosis Genetics Resource	606	0	0	0	0	2
GOLDN	893	phs001359	Genetics of Lipid Lowering Drugs and Diet Network	0	0	888	0	0	5
MGH_AF	916	phs001062	Massachusetts General Hospital (MGH) Atrial Fibrillation Study	0	0	893	13	10	0
BAGS	950	phs001143	The Genetics and Epidemiology of Asthma in Barbados	944	0	0	0	0	6
VU_AF	1021	phs001032	The Vanderbilt Atrial Fibrillation Registry	43	5	963	4	1	5
Amish	1027	phs000956	Genetics of Cardiometabolic Health in the Amish	0	0	1025	0	0	2
CRA	1042	phs000988	The Genetic Epidemiology of Asthma in Costa Rica	0	0	0	1040	0	2
GENOA	1070	phs001345	Genetic Epidemiology Network of Arteriopathy	1069	0	0	0	0	1
Mayo_VTE	1251	phs001402	Mayo Clinic Whole Genome Sequencing of Venous Thromboembolism	3	1	1180	7	13	47
SAFS	1480	phs001215	San Antonio Family Heart Study	0	0	0	1479	0	1
GeneSTAR	1635	phs001218	Genetic Study of Atherosclerosis Risk	726	0	907	0	0	2

GenSalt	1686	phs001217	Genetic Epidemiology Network of Salt Sensitivity	0	1680	0	0	0	6
HyperGEN	1753	phs001293	Hypertension Genetic Epidemiology Network (HyperGEN) - Genetics of Left Ventricular (LV) Hypertrophy	1751	1	0	0	0	1
JHS	3082	phs000964	The Jackson Heart Study	3074	0	0	0	0	8
ARIC	3589	phs001211	Atherosclerosis Risk in Communities Study	215	0	3244	0	0	130
FHS	3734	phs000974	Whole Genome Sequencing and Related Phenotypes in the Framingham Heart Study	2	0	3680	11	30	11
MESA	4861	phs001416	Multi-Ethnic Study of Atherosclerosis (MESA) and MESA Family AA-CAC	1665	547	1685	934	0	30
COPDGene	8486	phs000951	Genetic Epidemiology of COPD (COPDGene) in the TOPMed Program	2696	0	5751	0	0	39
WHI	10019	phs001237	Women's Health Initiative	1307	178	8151	273	72	38

Note that some listed studies are part of multiple TOPMed projects. For example, MESA includes both samples from the overall MESA project and MESA Family samples included in a project focused on coronary artery calcification in African Americans (AA-CAC), and ARIC includes samples from projects focused on venous thromboembolism (VTE) and atrial fibrillation (AFGen).
