# Supplementary material for: Use of >100,000 NHLBI Trans-Omics for Precision Medicine (TOPMed) Consortium whole genome sequences improves imputation quality and detection of rare variant associations in admixed African and Hispanic/Latino populations
Source: PLoS Genet. 2019 Dec 23;15(12):e1008500. doi: 10.1371/journal.pgen.1008500 (PMC6953885; doi:10.1371/journal.pgen.1008500)
Supplement: S3 Table — (PDF) [file pgen.1008500.s017.pdf]

S3 Table. Percentage and number of variants well-imputed with TOPMed freeze5b by chromosome in Jackson Heart Study (JHS) and Hispanic Community Health Study/Study of Latinos (HCHS/SOL)

	JHS		HCHS/SOL	
Chromosome	Percentage QC+ Variants	# QC+ Variants	Percentage QC+ Variants	# QC+ Variants
1	57.93%	4,122,556	65.40%	4,66,3289
2	58.31%	4,459,969	65.98%	5,055,379
3	58.45%	3,682,494	66.26%	4,182,405
4	59.20%	3,639,746	66.87%	4,118,691
5	58.89%	3,381,160	66.60%	3,830,843
6	59.24%	3,201,703	67.18%	3,638,786
7	58.67%	2,993,645	66.22%	3,385,271
8	58.91%	2,881,231	66.37%	3,252,246
9	58.05%	2,269,649	65.03%	2,547,068
10	58.85%	2,553,015	66.39%	2,886,243
11	58.51%	2,550,191	66.05%	2,885,087
12	58.39%	2,440,861	65.85%	2,758,834
13	58.84%	1,855,585	66.31%	2,095,287
14	58.55%	1,643,902	66.21%	1,862,507
15	58.56%	1,508,698	64.92%	1,676,421
16	56.92%	1,644,910	61.96%	1,794,239
17	57.36%	1,460,572	61.59%	1,5713,46
18	58.74%	1,457,749	66.29%	1,648,549
19	56.81%	1,126,064	61.04%	1,212,545
20	58.01%	1,185,170	62.61%	1,281,150
21	58.79%	693,483	62.00%	733,082
22	56.65%	715,169	60.59%	765,926
Total	58.44%	51,467,522	65.56%	57,845,194

Percentage QC+ Variants, percent of variants that were well-imputed for each chromosome and genome-wide; #QC+ Variants, number of variants that were well imputed. Post imputation quality control was carried out in seven MAF categories separately: <.05%, .05-.2%, .2-.5%, .5-1%, 1-3%, 3-5%, and >5%. In each MAF category, an estimated R^2 threshold (standard imputation software metric calculated based on the ratio of observed variance in imputed dosages over expected variance based on allele frequencies) was selected to ensure variants above the threshold have an average estimated R^2 of at least 0.8. These variants constitute the well imputed variants.
