# Supplementary material for: Use of >100,000 NHLBI Trans-Omics for Precision Medicine (TOPMed) Consortium whole genome sequences improves imputation quality and detection of rare variant associations in admixed African and Hispanic/Latino populations
Source: PLoS Genet. 2019 Dec 23;15(12):e1008500. doi: 10.1371/journal.pgen.1008500 (PMC6953885; doi:10.1371/journal.pgen.1008500)
Supplement: S4 Table — (PDF) [file pgen.1008500.s018.pdf]

S4 Table. Imputation quality for variants with a minor allele count between 11 and 20 in Jackson Heart Study (JHS)

JHS MAC	#Variants	#QC+	avgMAC	avgMAC_QC+	avgEstR^2	avgTrueR^2
11	623,867	564,102	180.4	181.8	93.2%	89.8%
12	547,859	495,767	196.9	198.6	93.3%	90.1%
13	491,677	445,126	213.8	215.9	93.5%	90.4%
14	441,924	400,220	228.6	230.5	93.6%	90.7%
15	407,835	369,084	244.8	246.6	93.8%	91.0%
16	371,311	336,419	260.3	262.4	93.9%	91.2%
17	341,915	309,949	275.3	277.6	94.0%	91.3%
18	319,797	290,096	291.6	293.6	94.1%	91.5%
19	296,527	268,825	305.6	308.2	94.2%	91.6%
20	278,642	252,898	322.9	326.2	94.3%	91.8%

MAC, minor allele count; #Variants, total number of variants with a given MAC in JHS which overlapped with the TOPMed freeze 5b reference panel; QC+, number of these variants which passed imputation quality control; avgMAC the average minor allele count in the (TOPMed freeze 5b minus JHS) reference panel of these variants; avgMAC QC+, the average minor allele count in the (TOPMed freeze 5b minus JHS) reference panel of variants which passed imputation quality control; avgEstR^2, average estimated R^2 for imputed variants (standard imputation software metric calculated based on the ratio of observed variance in imputed dosages over expected variance based on allele frequencies); avgTrueR^2, average true squared Pearson correlation between imputed genotypes and genotypes from available whole genome sequencing data
