# Supplementary material for: Use of >100,000 NHLBI Trans-Omics for Precision Medicine (TOPMed) Consortium whole genome sequences improves imputation quality and detection of rare variant associations in admixed African and Hispanic/Latino populations
Source: PLoS Genet. 2019 Dec 23;15(12):e1008500. doi: 10.1371/journal.pgen.1008500 (PMC6953885; doi:10.1371/journal.pgen.1008500)
Supplement: S5 Table — (PDF) [file pgen.1008500.s019.pdf]

S5 Table. Imputation quality for overall reference panel rare variants (20 or less MAC in TOPMed freeze 5b) in Jackson Heart Study (JHS)

			Any dosage sum ≥0			Any dosage sum ≥1			Any dosage sum ≥2			Any dosage sum ≥3		
TOPMed MAC	#Variants	#JHSpolymorphic	#QC+	avgEstR^2	avgTrueR^2	#QC+	avgEstR^2	avgTrueR^2	#QC+	avgEstR^2	avgTrueR^2	#QC+	avgEstR^2	avgTrueR^2
5	1,801,185	645,398	169,108	92.0%	85.6%	159,273	93.06%	88.00%	98,981	94.49%	93.03%	30,175	94.77%	90.46%
6	8,052,332	603,377	855,277	92.3%	87.3%	824,205	93.04%	88.87%	595,623	94.26%	92.88%	264,790	94.92%	92.15%
7	5,981,681	572,868	788,762	92.1%	87.0%	764,166	92.78%	88.35%	569,639	93.95%	92.29%	276,453	94.64%	92.03%
8	4,613,995	555,344	721,673	92.0%	86.6%	701,995	92.55%	87.78%	534,958	93.70%	91.66%	275,721	94.42%	91.73%
9	3,679,066	448,707	663,562	91.8%	86.4%	647,710	92.37%	87.43%	505,090	93.48%	91.12%	272,700	94.23%	91.35%
10	3,005,779	480,984	610,803	91.7%	86.2%	597,852	92.19%	87.10%	474,646	93.28%	90.62%	266,920	94.04%	91.03%
11	2,509,642	490,270	565,012	91.6%	86.1%	554,749	92.06%	86.89%	448,787	93.09%	90.16%	262,809	93.82%	90.63%
12	2,138,284	469,763	527,820	91.6%	86.1%	519,472	91.99%	86.83%	427,565	92.95%	89.90%	260,357	93.74%	90.49%
13	1,844,905	349,904	491,226	91.6%	86.0%	484,202	91.89%	86.65%	405,189	92.81%	89.56%	255,956	93.60%	90.24%
14	1,609,818	310,436	459,881	91.5%	86.1%	454,104	91.80%	86.64%	385,037	92.69%	89.34%	252,273	93.47%	90.04%
15	1,418,779	297,818	430,280	91.5%	86.0%	425,376	91.79%	86.55%	366,208	92.61%	89.08%	247,056	93.36%	89.84%
16	1,264,702	332,157	402,899	91.5%	86.1%	399,007	91.75%	86.57%	347,355	92.54%	88.90%	241,833	93.27%	89.65%
17	1,135,389	291,007	379,764	91.5%	86.1%	376,368	91.73%	86.49%	331,789	92.47%	88.63%	237,388	93.20%	89.40%
18	1,026,668	274,915	357,541	91.5%	86.2%	354,626	91.71%	86.58%	316,092	92.39%	88.57%	231,997	93.08%	89.34%
19	933,486	222,965	338,078	91.5%	86.2%	335,677	91.70%	86.55%	301,867	92.35%	88.38%	225,904	93.03%	89.20%
20	856,539	229,124	321,114	91.5%	86.4%	318,852	91.71%	86.68%	289,242	92.33%	88.37%	221,679	92.97%	89.14%

MAC, minor allele count; #Variants, total number of variants with a given MAC in the (TOPMed freeze 5b minus JHS) reference panel; #JHS polymorphic, number of these variants that are polymorphic in JHS; #QC+, number of polymorphic JHS variants which passed imputation quality control; avgEstR^2, average estimated R^2 for imputed variants (standard imputation software metric calculated based on the ratio of observed variance in imputed dosages over expected variance based on allele frequencies); avgTrueR^2, average true squared Pearson between imputed genotypes and genotypes from available whole genome sequencing data. These metrics are listed for a JHS dosage sum ≥0, a dosage sum ≥1, a dosage sum ≥2, and a dosage sum ≥3.
