# Supplementary material for: Use of >100,000 NHLBI Trans-Omics for Precision Medicine (TOPMed) Consortium whole genome sequences improves imputation quality and detection of rare variant associations in admixed African and Hispanic/Latino populations
Source: PLoS Genet. 2019 Dec 23;15(12):e1008500. doi: 10.1371/journal.pgen.1008500 (PMC6953885; doi:10.1371/journal.pgen.1008500)
Supplement: S6 Table — (PDF) [file pgen.1008500.s020.pdf]

S6 Table. Imputation quality for rare variants (20 or less MAC) in Hispanic Community Health Study/Study of Latinos (HCHS/SOL)

HCHS/SOL MAC	#Variants	#QC+	avgMAC	avgMAC QC+	avgR^2	#MEGA	#MEGA_QC+	avgMAC (MEGA)	avgMAC_QC+ (MEGA)	avgEstR^2	avgTrue R^2 (MEGA)
1	13,474,575	6,105,358	13.3	14.0	75.9%	26059	10666	17.2	17.9	74.7%	67.1%
2	7,164,057	5,603,722	18.3	18.2	73.5%	16713	12529	22.8	22.6	71.1%	63.7%
3	4,642,367	4,087,905	23.1	22.9	75.5%	12307	10635	28.4	28.0	72.7%	64.7%
4	3,337,192	3,073,145	27.7	27.4	77.5%	9530	8612	33.1	32.6	75.0%	65.3%
5	2,554,367	2,403,752	32.3	31.9	79.1%	7862	7389	37.1	36.9	77.0%	67.2%
6	2,041,019	1,947,929	36.9	36.6	80.2%	6644	6349	41.7	41.5	78.4%	68.1%
7	1,690,458	1,628,881	41.8	41.6	81.2%	5484	5282	46.1	45.9	80.2%	70.4%
8	1,428,173	1,385,141	46.8	46.6	82.0%	4821	4678	50.0	49.9	80.7%	71.0%
9	1,234,675	1,202,963	50.9	50.8	82.7%	4210	4123	54.8	54.5	81.4%	71.9%
10	1,078,841	1,055,149	55.9	55.9	83.2%	3753	3682	60.1	60.1	82.3%	72.9%
11	763,500	749,345	60.1	60.1	84.4%	2562	2527	63.1	63.1	83.6%	74.7%
12	873,034	857,790	63.9	63.8	83.9%	3106	3064	67.7	67.7	83.7%	74.3%
13	786,768	774,382	68.9	69.0	84.3%	2754	2714	73.4	73.6	83.6%	74.4%
14	711,019	700,954	73.8	73.8	84.7%	2572	2544	77.5	77.5	84.2%	76.2%
15	653,169	644,826	78.0	77.3	85.1%	2370	2344	80.1	80.2	84.6%	76.1%
16	599,138	592,233	84.2	83.7	85.4%	2162	2138	85.9	85.7	84.9%	76.7%
17	554,471	548,484	88.5	87.3	85.7%	2003	1983	90.9	90.7	84.8%	76.6%
18	514,587	509,546	94.5	93.5	86.0%	1860	1850	96.3	96.5	85.3%	77.0%
19	478,973	474,690	100.7	98.3	86.3%	1774	1756	101.9	102.1	85.9%	77.8%
20	447,355	443,687	105.1	103.7	86.5%	1681	1670	102.5	102.6	85.9%	76.8%

MAC, minor allele count; #Variants, total number of variants with a given MAC in HCHS/SOL; # QC+, number of these variants that are well imputed in HCHS/SOL; avgMAC, the average minor allele count in the TOPMed freeze 5b reference panel of these variants; avgMAC QC+, the average minor allele count in the TOPMed freeze 5b reference panel of variants which passed imputation quality control; avgR^2, average estimated R^2 for imputed variants; #MEGA, number of variants with this MAC overlapping the MEGA array; #MEGA_QC+, number of variants with this MAC overlapping the MEGA array which passed imputation quality control; avgMAC (MEGA), the average minor allele count in the TOPMed freeze 5b reference panel of these variants which overlap MEGA; avgMAC_QC+ (MEGA), the average minor allele count in the TOPMed freeze 5b reference panel of variants which passed imputation quality control which overlap MEGA; avgEstR^2 , average estimated R^2 for imputed variants which overlap MEGA (standard imputation software metric calculated based on the ratio of observed variance in imputed dosages over expected variance based on allele frequencies); avgTrueR^2, average true squared Pearson correlation between imputed genotypes and genotypes from available direct genotyping
