# Supplementary material for: Use of >100,000 NHLBI Trans-Omics for Precision Medicine (TOPMed) Consortium whole genome sequences improves imputation quality and detection of rare variant associations in admixed African and Hispanic/Latino populations
Source: PLoS Genet. 2019 Dec 23;15(12):e1008500. doi: 10.1371/journal.pgen.1008500 (PMC6953885; doi:10.1371/journal.pgen.1008500)
Supplement: S7 Table — (PDF) [file pgen.1008500.s021.pdf]

S7 Table. Imputation quality for overall reference panel rare variants (20 or less MAC) in TOPMed freeze 5b in Hispanic Community Health Study/Study of Latinos (HCHS/SOL)

TOPMed MAC	#Variants	#HCHS/SOL polymorphic	#QC+	avgR^2	#MEGA	#MEGA_QC+	avgEstR^2	avgTrueR^2
5	1,818,306	830,932	763,653	80.6%	1172	1071	76.4%	54.0%
6	8,127,408	2,967,730	2,476,304	76.0%	3603	3082	75.1%	56.7%
7	6,038,334	2,503,086	2,091,439	76.2%	3376	2823	74.8%	56.6%
8	4,657,557	2,143,361	1,792,887	76.5%	3444	2917	75.3%	58.1%
9	3,714,229	1,863,641	1,563,270	76.7%	3202	2678	75.3%	60.1%
10	3,034,091	1,640,207	1,378,429	77.0%	3199	2672	75.0%	59.4%
11	2,533,108	1,458,534	1,230,608	77.2%	3103	2594	76.3%	62.3%
12	2,158,188	1,310,316	1,109,078	77.5%	2829	2341	75.8%	62.2%
13	1,862,539	1,185,008	1,005,752	77.7%	2756	2267	75.8%	64.0%
14	1,625,131	1,077,115	919,620	77.9%	2759	2297	75.9%	63.3%
15	1,432,061	983,423	841,897	78.1%	2665	2256	76.3%	65.3%
16	1,276,423	905,196	777,668	78.4%	2558	2119	76.2%	66.1%
17	1,146,048	835,581	720,865	78.5%	2369	1963	77.2%	65.6%
18	1,036,400	774,868	671,247	78.7%	2409	1981	77.2%	67.5%
19	942,107	719,813	626,646	79.0%	2247	1902	77.0%	66.6%
20	864,490	673,914	588,149	79.1%	2146	1828	76.7%	66.5%

MAC, minor allele count; #Variants, total number of variants with a given MAC in TOPMed freeze 5b; #HCHS/SOL polymorphic, number of these variants polymorphic in HCHS/SOL imputation results; #QC+, number of these variants that are well imputed in HCHS/SOL; avgR^2, average estimated R^2 for imputed variants; #MEGA, number of variants with this MAC overlapping the MEGA array; #MEGA_QC+, number of variants with this MAC overlapping the MEGA array which passed imputation quality control; avgEstR^2, average estimated R^2 for imputed variants which overlap MEGA (standard imputation software metric calculated based on the ratio of observed variance in imputed dosages over expected variance based on allele frequencies); avgTrueR^2, true correlation between imputed genotypes and genotypes from available direct genotyping
