# Supplementary material for: Use of >100,000 NHLBI Trans-Omics for Precision Medicine (TOPMed) Consortium whole genome sequences improves imputation quality and detection of rare variant associations in admixed African and Hispanic/Latino populations
Source: PLoS Genet. 2019 Dec 23;15(12):e1008500. doi: 10.1371/journal.pgen.1008500 (PMC6953885; doi:10.1371/journal.pgen.1008500)
Supplement: S8 Table — (PDF) [file pgen.1008500.s022.pdf]

S8 Table. Imputation quality for rare and low frequency variants estimated to be well imputed in Table 1 stratified by regional background in in the Hispanic Community Health Study/Study of Latinos (HCHS/SOL).

avgTrueR^2	Well imputed variants with MAF < 0.5%						Well imputed variants with MAF < 0.05%					
	Central American	Cuban	Dominican	Mexican	Puerto Rican	South American	Central American	Cuban	Dominican	Mexican	Puerto Rican	South American
TOPMed Freeze 5b	89.26%	86.68%	90.59%	83.26%	91.43%	90.08%	84.70%	76.30%	85.08%	69.07%	84.23%	81.74%
1000G	80.64%	80.25%	82.03%	75.15%	83.50%	80.90%	74.65%	80.09%	77.25%	62.78%	77.70%	66.89%
HRC	65.18%	66.00%	65.58%	62.70%	66.79%	65.81%	56.67%	59.00%	67.48%	59.75%	66.83%	59.73%

avgTrueR^2, true correlation between imputed genotypes and genotypes from available whole genome sequencing or genotyping data.
Imputation reference panels: TOPMed freeze 5b, 1000 Genomes Phase 3 (1000G) and Haplotype Reference Consortium (HRC).
