# Supplementary material for: Use of >100,000 NHLBI Trans-Omics for Precision Medicine (TOPMed) Consortium whole genome sequences improves imputation quality and detection of rare variant associations in admixed African and Hispanic/Latino populations
Source: PLoS Genet. 2019 Dec 23;15(12):e1008500. doi: 10.1371/journal.pgen.1008500 (PMC6953885; doi:10.1371/journal.pgen.1008500)
Supplement: S9 Table — (PDF) [file pgen.1008500.s023.pdf]

S9 Table. Demographics, hematological traits, and number of ancestry principal components adjusted for in association analysis models for cohorts imputed to TOPMed freeze 5b reference panel.

					Age	Hemoglobin (g/dL)		Hematocrit (%)		White blood cell count ($x10^9$/L)		Hematology Methods
Cohort	Self-Identified Ancestry	n	PCs	% female	Mean (SD)	Mean (SD)	n	Mean (SD)	n	Mean (SD)	n	
GERA	Hispanic/Latino	5783	6	63.74%	56.54 (14.5)	13.82 (1.38)	5783	41.33 (3.67)	5783	6.79 (2.24)	5783	Standard clinical methods (biobank)
GERA	African American	2246	6	62.51%	59.57 (13.42)	13.26 (1.41)	2246	40.13 (3.83)	2246	6.28 (2.29)	2246	Standard clinical methods (biobank)
WHI[1]	Hispanic/Latino	1081	10	100%	60.20 (6.85)	13.46 (0.92)	1081	40.05 (2.72)	1081	6.17 (1.55)	1079	(1)
WHI[1]	African American	1366	10	100%	60.75 (7.12)	12.86 (1.09)	1366	38.96 (3.15)	1365	5.65 (2.55)	1366	(1)
WHI[2]	Hispanic/Latino	3237	10	100%	60.31 (6.64)	13.40 (1.05)	3234	39.87 (2.91)	3232	6.04 (1.91)	3235	(1)
WHI[2]	African American	4658	10	100%	59.74 (6.50)	12.89 (1.05)	4648	39.00 (3.03)	4646	5.63 (1.81)	4652	(1)
WHI[3]	African American	2470	10	100%	65.03 (6.56)	12.92 (1.03)	2469	39.08 (3.13)	2467	5.64 (2.31)	2468	(1)
HCHS/SOL	Hispanic/Latino	11588	10	58.41%	46.05 (13.83)	13.80 (1.51)	11588	42.12 (4.07)	11588	6.50 (1.92)	10937	(2, 3)
CARDIA	African American	1619	10	58.77%	24.43 (3.79)	13.81 (1.53)	1618	41.20 (4.55)	1619	5.64 (1.36)	1619	(4)
UK Biobank	African	6762	10	57.07%	51.88 (8.06)	13.51 (1.42)	6762	40.07 (4.03)	6762	5.72 (1.63)	6762	(5)
ARIC	African American	2392	10	62.39%	53.48 (5.80)	13.23 (1.47)	2392	40.36 (4.33)	2392	5.40 (1.37)	2392	(6, 7)

ARIC, Atherosclerosis Risk in Communities; CARDIA, Coronary Artery Risk Development in Young Adults; HCHS/SOL, Hispanic Community Health Study/Study of Latinos; GERA, Resource for Genetic Epidemiology Research on Aging; PCs, ancestry principal components; SD, standard deviation; WHI[1], Women's Health Initiative (MEGA only); WHI[2], Women's Health Initiative (MEGA and Affymetrix 6.0); WHI[3], Women's Health Initiative (Affymetrix 6.0 only)
