# Supplementary material for: Use of >100,000 NHLBI Trans-Omics for Precision Medicine (TOPMed) Consortium whole genome sequences improves imputation quality and detection of rare variant associations in admixed African and Hispanic/Latino populations
Source: PLoS Genet. 2019 Dec 23;15(12):e1008500. doi: 10.1371/journal.pgen.1008500 (PMC6953885; doi:10.1371/journal.pgen.1008500)
Supplement: S10 Table — (PDF) [file pgen.1008500.s024.pdf]

S10 Table. Demographics, hematological traits, and number of ancestry principal components adjusted for in association analysis for African American cohorts with sequencing and hematological trait data from TOPMed freeze 5b

Cohort	n	PCs	% female	Age	Hemoglobin (g/dL)		Hematocrit (%)		White blood cell count ($x10^9/L$)		Hematology Methods
					Mean (SD)	n	Mean (SD)	n	Mean (SD)	n	
ARIC	210	10	59.05%	54.63 (5.87)	13.13 (1.65)	210	40.20 (4.94)	210	6.15 (2.08)	210	(6, 7)
GeneSTAR	694	10	63.40%	42.92 (12.38)	12.96 (1.35)	682	39.58 (3.86)	694	5.99 (1.94)	694	(8)
JHS	2936	10	62.13%	54.65 (12.84)	13.06 (1.50)	2932	39.31 (4.28)	2933	5.63 (1.87)	2627	(9)
WHI	1296	10	100.00%	63.60 (6.97)	13.00 (1.40)	1296	39.40 (4.34)	1296	5.66 (1.81)	1295	(1)
COPDGene	1353	10	49.74%	59.84 (7.07)	13.51 (1.55)	1353	41.13 (4.36)	1353	6.68 (2.31)	1353	(10, 11)
MESA	564	10	52.30%	69.46 (9.05)	13.09 (1.43)	564	39.40 (3.92)	564	5.75 (1.86)	564	(6, 12)

ARIC, Atherosclerosis Risk in Communities; COPDGene, Genetic Epidemiology of Chronic Obstructive Pulmonary Disease; JHS, Jackson Heart Study; MESA, Multi-Ethnic Study of Atherosclerosis; SD, standard deviation
