# Supplementary material for: Use of >100,000 NHLBI Trans-Omics for Precision Medicine (TOPMed) Consortium whole genome sequences improves imputation quality and detection of rare variant associations in admixed African and Hispanic/Latino populations
Source: PLoS Genet. 2019 Dec 23;15(12):e1008500. doi: 10.1371/journal.pgen.1008500 (PMC6953885; doi:10.1371/journal.pgen.1008500)
Supplement: S11 Table — (PDF) [file pgen.1008500.s025.pdf]

S11 Table. Overall counts for variants replicated in TOPMed freeze 5b imputed cohorts.

	# Hispanic/Latino reported variants[1]	# in freeze5	#w/*P*<5x10^-8	#w/P<5x10^-6	#w/P<0.05	Variants not in TOPMed freeze 5b
HCT	4	3	3	3	3	rs334
HGB	4	3	2	3	3	esv2676630/3.8kbdel
WBC	5	4	2	3	4	rs2524079

	#African ancestry reported variants[2]	# in freeze5	#w/*P*<5x10^-8	#w/P<5x10^-6	#w/P<0.05	Variants not in TOPMed freeze 5b
HCT	3	1	0	1	1	rs334,rs2213169
HGB	6	6	6	6	6	
WBC	15	14	13	13	14	rs2518564

1: variants with p-value <5x10^-8, previously reported from Hispanic/Latino samples

2: variants with p-value <5x10^-8, previously reported from African American samples
