# Supplementary material for: Use of >100,000 NHLBI Trans-Omics for Precision Medicine (TOPMed) Consortium whole genome sequences improves imputation quality and detection of rare variant associations in admixed African and Hispanic/Latino populations
Source: PLoS Genet. 2019 Dec 23;15(12):e1008500. doi: 10.1371/journal.pgen.1008500 (PMC6953885; doi:10.1371/journal.pgen.1008500)
Supplement: S12 Table — (PDF) [file pgen.1008500.s026.pdf]

S12 Table. Results for previously identified variants in African ancestry and Hispanic/Latino populations in TOPMed freeze 5b imputed samples (included cohorts detailed in S1 and S8 Tables).

SNP	Pos:Ref:Alt	Effect Allele	Ancestry	Trait	EAF Range	R^2 Range	previous β	previous P-value	β	P-value	β (adjusted for duffy)	P-value (adjusted for duffy)	Reference
**rs3754140**	1:214003037:T:C	C	Hispanic/Latino	HCT	33.49%-39.00%	0.987-0.995	0.24	5.70E-08	0.061	3.65E-09	NA	NA	(2)
**rs17034641**	2:46145505:G:A	A	Hispanic/Latino	HCT	13.56%-14.39%	0.988-0.998	-0.360	2.69E-09	-0.080	9.39E-09	NA	NA	(2)
**rs334**	11:5227002:A:T	T	Hispanic/Latino	HCT	not available in TOPMed5b	NA	1.32	1.30E-10	NA	NA	NA	NA	(2)
**rs855791**	22:37066896:A:G	G	Hispanic/Latino	HCT	54.29%-55.92%	0.939-0.997	0.380	1.10E-10	0.064	1.72E-10	NA	NA	(2)
**rs13008603**	2:46128709:C:A	A	AA	HCT	12.78%-17.07%	0.950-0.999	-0.277	4.09E-09	-0.068	3.65E-07	NA	NA	(13)
**rs334**	11:5227002:A:T	T	AA	HCT	not available in TOPMed5b	NA	-0.688	5.70E-11	NA	NA	NA	NA	(14)
**rs2213169**	11:5281833:C:T	T	AA	HCT	not available in TOPMed5b	NA	-0.447	4.94E-11	NA	NA	NA	NA	(15)
**rs17034641**	2:46145505:G:A	A	Hispanic/Latino	HGB	13.56%-14.39%	0.988-0.998	-0.120	3.20E-08	-0.073	1.55E-07	NA	NA	(2)
**rs2032451**	6:26091942:G:T	T	Hispanic/Latino	HGB	11.73%-13.65%	0.999-0.999	0.120	3.10E-08	0.102	1.82E-11	NA	NA	(2)
**esv2676630/3.8kbdel**	16:173448	NA	Hispanic/Latino	HGB	not available in TOPMed5b	NA	-0.46	1.00E-32	NA	NA	NA	NA	(2)
**rs855791**	22:37066896:A:G	G	Hispanic/Latino	HGB	54.29%-55.92%	0.939-0.997	0.150	6.00E-23	0.109	2.70E-28	NA	NA	(2)
**rs2562181**	16:91661:C:T	T	AA	HGB	20.22%-35.93%	0.955-0.990	-0.098	5.05E-14	-0.076	6.86E-13	NA	NA	(16)
**rs7203560**	16:134391:T:G	G	AA	HGB	4.35%-7.92%	0.949-0.999	-0.199	2.23E-08	-0.183	2.56E-22	NA	NA	(15)
**rs11863726**	16:180579:A:G	G	AA	HGB	31.14%-48.72%	0.824-0.981	-0.130	8.90E-11	-0.104	5.71E-24	NA	NA	(14)
**rs13335497**	16:260006:G:A	A	AA	HGB	6.03%-11.82%	0.914-0.994	-0.344	1.80E-21	-0.233	4.94E-48	NA	NA	(14)
**rs13335629**	16:260381:G:A	A	AA	HGB	6.79%-13.44%	0.915-0.997	-0.190	2.63E-23	-0.208	1.12E-43	NA	NA	(13)
**rs9924561**	16:264781:G:T	T	AA	HGB	5.95%-11.67%	0.908-0.999	-0.389	6.60E-24	-0.238	2.03E-49	NA	NA	(14)
**rs2814778**	1:159204893:T:C	C	Hispanic/Latino	WBC#	4.22%-14.53%	0.992-1.000	-0.104	5.68E-56	-0.373	1.78E-80	NA	NA	(3)
**rs114477531**	2:42919281:T:C	C	Hispanic/Latino	WBC#	0.80%-1.32%	0.931-0.997	-0.091	3.60E-08	-0.100	0.030	-0.099	0.032	(3)
**rs2524079**	6:31274397:G:A	A	Hispanic/Latino	WBC#	not available in TOPMed5b	NA	0.0213	1.50E-08	NA	NA	NA	NA	(3)
**rs2380606**	8:69828661:T:C	C	Hispanic/Latino	WBC#	47.77%-51.66%	0.980-0.999	-0.024	8.00E-10	-0.049	1.47E-06	-0.047	3.08E-06	(3)
**rs2227336**	17:40018602:T:G	G	Hispanic/Latino	WBC#	33.52%-35.60%	0.991-1.000	0.026	8.80E-11	0.096	1.18E-19	0.096	8.14E-20	(3)
**rs3811035**	1:157515771:G:A	A	AA	WBC#	15.62%-36.92%	0.937-0.989	0.187	8.01E-09	0.279	1.41E-82	0.022	0.130	(17)
**rs945635**	1:157700500:C:G	G	AA	WBC#	66.75%-86.52%	0.950-0.993	-0.210	2.46E-10	-0.253	9.56E-64	-0.014	0.347	(17)
**rs4971154**	1:157802090:C:T	T	AA	WBC#	11.32%-30.61%	0.955-0.996	0.218	3.35E-10	0.247	2.21E-55	-0.002	0.877	(17)

**rs6427419**	1:158088319:C:A	A	AA	WBC#	10.06%-28.74%	0.965-0.999	0.319	3.17E-17	0.314	7.22E-75	-0.017	0.336	(17)
**rs4657616**	1:159001296:A:G	G	AA	WBC#	6.28%-21.82%	0.914-0.995	0.061	5.48E-47	0.443	3.19E-103	0.023	0.291	(15)
**rs1057024**	1:159020457:A:G	G	AA	WBC#	45.28-71.19%	0.953-0.999	-0.185	6.71E-14	-0.227	1.47E-91	-0.029	0.013	(17)
**rs2518564**	1:159092646:G:A	A	AA	WBC#	not available in TOPMed5b	NA	0.191	1.39E-149	NA	NA	NA	NA	(18)
**rs2814778**	1:159204893:T:C	C	AA	WBC#	46.35%-83.45%	0.913-1.000	-0.533	4.75152E-309	-0.758	0.00E+00	NA	NA	(19)
**rs12075**	1:159205564:G:A	A	AA	WBC#	77.24%-91.97%	0.812-0.973	-0.371	2.26E-17	-0.434	2.31E-101	0.024	0.284	(17)
**rs2808661**[1]	1:159588468:A:G	G	AA	WBC#	88.86%-96.71%	0.903-1.000	-0.353	1.47E-09	-0.288	1.24E-21	-0.030	0.309	(17)
**rs35940156**	2:135817730:C:T	T	AA	WBC#	1.46%-2.64%	0.988-1.000	0.189	4.27E-08	0.223	3.15E-12	0.217	7.87-13	(19)
**rs35837297**	2:135836869:T:C	C	AA	WBC#	1.50%-2.77%	0.987-1.000	0.195	1.54E-08	0.223	2.09E-12	0.212	1.62E-12	(19)
**rs9131**	4:74097332:C:T	T	AA	WBC#	21.11%-40.13%	0.973-1.000	-0.023	1.58E-08	-0.049	5.49E-05	-0.040	5.13E-04	(20)
**rs445**	7:92779056:C:T	T	AA	WBC#	15.64%-19.92%	0.932-0.999	-0.067	1.40E-08	-0.069	2.97E-08	-0.068	7.42E-09	(19)
**rs4794822**	17:40000459:C:T	T	AA	WBC#	30.89%-33.95%	0.981-1.000	0.059	1.31E-08	0.064	2.54E-09	0.064	3.46E-10	(19)

AA, African ancestry, HCT, hematocrit, HGB, hemoglobin, WBC#, white blood cell count, duffy, Duffy variant (rs2814778)

1: Association results do not include UK Biobank cohort, as estimated R^2 for rs2808661 was 0.757. The estimated R^2 of all other variants in this table exceeded 0.8 for all cohorts of the relevant ancestry.
