# Supplementary material for: Use of >100,000 NHLBI Trans-Omics for Precision Medicine (TOPMed) Consortium whole genome sequences improves imputation quality and detection of rare variant associations in admixed African and Hispanic/Latino populations
Source: PLoS Genet. 2019 Dec 23;15(12):e1008500. doi: 10.1371/journal.pgen.1008500 (PMC6953885; doi:10.1371/journal.pgen.1008500)
Supplement: S13 Table — (PDF) [file pgen.1008500.s027.pdf]

S13 Table. Imputation of novel variants identified with TOPMed freeze 5b-based imputation using current widely used reference panels from the Haplotype Reference Consortium (HRC) and 1000 Genomes Phase 3, as well as subsequent association analysis results for cohorts where the variants were well-imputed (R^2>0.8).

rsID	Reference	Pos:Ref:Alt	Effect Allele	Non-effect allele	Ancestry	Trait	Imputed MAF	R^2 Range	β	P-value
rs33930165	1000G	11:5248233:C:T	T	C	AA	WBC	0.012	0.126-0.975	0.324	7.52E-06
rs33930165	HRC	11:5248233:C:T	T	C	AA	WBC	0.017	0.456-0.961	0.225	1.71E-07
rs11549407	HRC	11:5248004:G:A	A	G	HL	HCT	0.000602	0.413-0.987	-2.034	4.77E-06
rs11549407	HRC	11:5248004:G:A	A	G	HL	HGB	0.000602	0.413-0.987	-2.340	1.33E-07

HCT, hematocrit; HGB, hemoglobin; WBC, white blood cell count; MAF, minor allele frequency; AA, African ancestry; HL, Hispanic/Latino. While rare variant rs11549407 is present in the HRC reference panel, individual level genotypes are not available for HRC. We are thus not able to examine which individuals (for example from the Sardinian population) are contributing to the imputation of this variant using the HRC reference panel.
