# Supplementary material for: Use of >100,000 NHLBI Trans-Omics for Precision Medicine (TOPMed) Consortium whole genome sequences improves imputation quality and detection of rare variant associations in admixed African and Hispanic/Latino populations
Source: PLoS Genet. 2019 Dec 23;15(12):e1008500. doi: 10.1371/journal.pgen.1008500 (PMC6953885; doi:10.1371/journal.pgen.1008500)
Supplement: S14 Table — (PDF) [file pgen.1008500.s028.pdf]

S14 Table. Estimated imputation quality for rs33930165 and rs11549407 using 1000G phase 3 and Haplotype Reference Consortium (HRC) as references.

rsID	Pos:Ref:Alt	Ancestry	Imputation Reference	Cohort	EstR^2	Genotyped
rs33930165	11:5248233:C:T	AA	1000G	WHI[1]	0.975	Genotyped
				WHI[2]	0.975	Genotyped
				WHI[3]	0.741	Imputed only
				ARIC	0.126	Imputed only
				GERA	0.825	Imputed only
				UK Biobank	0.709	Genotyped
				CARDIA	0.609	Imputed only
rs33930165	11:5248233:C:T	AA	HRC	WHI[1]	0.961	Genotyped
				WHI[2]	0.961	Genotyped
				WHI[3]	0.808	Imputed only
				ARIC	0.456	Imputed only
				GERA	0.860	Imputed only
				UK Biobank	0.845	Imputed only
				CARDIA	0.812	Imputed only
rs11549407	11:5248004:G:A	Hispanic/Latino	HRC	WHI[1]	0.987	Genotyped
				WHI[2]	0.954	Genotyped
				HCHS/SOL	0.523	Imputed only
				GERA	0.413	Imputed Only

EstR^2, estimated imputation quality, AA, African ancestry.

Imputed dosages were used for association analyses, regardless of availability of genotype data.

WHI[1]: Women's Health Initiative (MEGA only)

WHI[2]: Women's Health Initiative (MEGA and Affymetrix 6.0)

WHI[3]: Women's Health Initiative (Affymetrix 6.0 only)

GERA: Resource for Genetic Epidemiology Research on Aging

ARIC: Atherosclerosis Risk in Communities Study

HCHS/SOL: Hispanic Community Health Study/Study of Latinos

CARDIA: Coronary Artery Risk Development in Young Adults
