# Supplementary material for: Use of >100,000 NHLBI Trans-Omics for Precision Medicine (TOPMed) Consortium whole genome sequences improves imputation quality and detection of rare variant associations in admixed African and Hispanic/Latino populations
Source: PLoS Genet. 2019 Dec 23;15(12):e1008500. doi: 10.1371/journal.pgen.1008500 (PMC6953885; doi:10.1371/journal.pgen.1008500)
Supplement: S15 Table — (PDF) [file pgen.1008500.s029.pdf]

S15 Table. Association statistics for the hemoglobin C variant (rs33930165, 11:5227003:C:T) with white blood cell subtypes, adjusting for age, sex, and ancestry principal components.

Cohorts	Trait	N	beta	*P*
TOPMed freeze 5b Imputed African Ancestry Cohorts	Total WBC	20804	0.349	8.77E-15
	Neutrophil	12863	0.163	0.003
	Lymphocyte	12863	0.313	5.39E-07
	Eosinophil	8636	0.010	0.194
	Basophil	8660	0.116	0.114
	Monocyte	12842	0.126	0.040
Sequenced African Ancestry Samples from TOPMed	Total WBC	6740	0.270	4.47E-04
	Neutrophil	4649	0.111	0.165
	Lymphocyte	5278	0.379	1.04E-05
	Eosinophil	4609	0.074	0.414
	Basophil	4470	0.131	0.160
	Monocyte	5253	0.188	0.026

All analyses are adjusted for the Duffy variant rs2814778, and rs334 minor allele homozygotes (who may have sickle cell anemia) are removed.
