# Supplementary material for: Use of >100,000 NHLBI Trans-Omics for Precision Medicine (TOPMed) Consortium whole genome sequences improves imputation quality and detection of rare variant associations in admixed African and Hispanic/Latino populations
Source: PLoS Genet. 2019 Dec 23;15(12):e1008500. doi: 10.1371/journal.pgen.1008500 (PMC6953885; doi:10.1371/journal.pgen.1008500)
Supplement: S16 Table — (PDF) [file pgen.1008500.s030.pdf]

S16 Table. White blood cell subtypes for cohorts imputed to TOPMed freeze 5b reference panel.

Cohort	Self-Identified Ancestry	Relative basophil count ($x10^9/L$)		Relative eosinophil count ($x10^9/L$)		Relative lymphocyte count ($x10^9/L$)		Relative monocyte count ($x10^9/L$)		Relative neutrophil count ($x10^9/L$)	
		Mean (SD)	n	Mean (SD)	n	Mean (SD)	n	Mean (SD)	n	Mean (SD)	n
GERA	Hispanic/Latino	NA	NA	NA	NA	2.03 (0.94)	4575	0.54 (0.2)	4575	4.08 (1.93)	4575
GERA	African American	NA	NA	NA	NA	2.08 (0.69)	1809	0.52 (0.22)	1809	3.54 (2.02)	1809
WHI[1]	Hispanic/Latino	0.04 (0.02)	57	0.20 (0.13)	57	1.88 (0.64)	57	0.58 (0.17)	57	3.89 (1.08)	57
WHI[1]	African American	0.03 (0.02)	96	0.17 (0.11)	97	1.89 (0.72)	98	0.52 (0.23)	98	2.92 (1.34)	98
WHI[2]	Hispanic/Latino	0.04 (0.02)	1087	0.19 (0.14)	1087	1.86 (1.01)	1093	0.54 (0.16)	1093	3.62 (1.33)	1093
WHI[2]	African American	0.03 (0.03)	1333	0.18 (0.12)	1332	1.93 (0.67)	1342	0.53 (0.18)	1342	3.25 (1.51)	1342
WHI[3]	African American	0.03 (0.03)	605	0.19 (0.16)	607	1.86 (0.78)	609	0.54 (0.21)	609	3.21 (1.49)	609
HCHS/SOL	Hispanic/Latino	NA	NA	NA	NA	2.13 (0.69)	10935	0.52 (0.23)	10935	3.64 (1.54)	10936
CARDIA	African American	NA	NA	NA	NA	NA	NA	NA	NA	NA	NA
UK Biobank	African	0.03 (0.05)	6737	0.15 (0.13)	6712	2.10 (0.79)	6738	0.39 (0.18)	6738	3.03 (1.23)	6738
ARIC	African American	NA	NA	NA	NA	2.06 (1.42)	2382	0.30 (1.73)	2361	2.56(1.63)	2382

GERA: Resource for Genetic Epidemiology Research on Aging
WHI[1]: Women's Health Initiative (MEGA only)
WHI[2]: Women's Health Initiative (MEGA and Affymetrix 6.0)
WHI[3]: Women's Health Initiative (Affymetrix 6.0 only)
HCHS/SOL: Hispanic Community Health Study/Study of Latinos
CARDIA: Coronary Artery Risk Development in Young Adults
ARIC: Atherosclerosis Risk in Communities
SD: standard deviation
