# Supplementary material for: Use of >100,000 NHLBI Trans-Omics for Precision Medicine (TOPMed) Consortium whole genome sequences improves imputation quality and detection of rare variant associations in admixed African and Hispanic/Latino populations
Source: PLoS Genet. 2019 Dec 23;15(12):e1008500. doi: 10.1371/journal.pgen.1008500 (PMC6953885; doi:10.1371/journal.pgen.1008500)
Supplement: S17 Table — (PDF) [file pgen.1008500.s031.pdf]

S17 Table. White blood cell subtypes for African American cohorts with sequencing and hematological trait data from TOPMed freeze 5b

| | Relative basophil count ($x10^9/L$) | | Relative eosinophil count ($x10^9/L$) | | Relative lymphocyte count ($x10^9/L$) | | Relative monocyte count ($x10^9/L$) | | Relative neutrophil count ($x10^9/L$) | |
|---|---|---|---|---|---|---|---|---|---|---|
| Cohort | Mean (SD) | n | Mean (SD) | n | Mean (SD) | n | Mean (SD) | n | Mean (SD) | n |
| ARIC | 0.04 (0.05) | 197 | 0.19 (0.25) | 197 | 2.25 (0.79) | 197 | 0.35 (0.18) | 197 | 3.27 (1.56) | 197 |
| GeneSTAR | NA | NA | NA | NA | 2.22 (0.71) | 628 | 0.31 (0.17) | 607 | NA | NA |
| JHS | 0.03 (0.02) | 2434 | 0.14 (0.12) | 2573 | 1.93 (0.66) | 2613 | 0.39 (0.15) | 2610 | 3.12 (1.40) | 2612 |
| WHI | NA | NA | NA | NA | NA | NA | NA | NA | NA | NA |
| COPDGene | 0.03 (0.04) | 1343 | 0.16 (0.14) | 1343 | 2.22 (087) | 1344 | 0.50 (0.20) | 1343 | 3.76 (1.86) | 1344 |
| MESA | 0.01 (0.03) | 496 | 0.17 (0.15) | 496 | 1.87 (0.61) | 496 | 0.43 (0.16) | 496 | 3.17 (1.38) | 496 |
