# Supplementary material for: Use of >100,000 NHLBI Trans-Omics for Precision Medicine (TOPMed) Consortium whole genome sequences improves imputation quality and detection of rare variant associations in admixed African and Hispanic/Latino populations
Source: PLoS Genet. 2019 Dec 23;15(12):e1008500. doi: 10.1371/journal.pgen.1008500 (PMC6953885; doi:10.1371/journal.pgen.1008500)
Supplement: S18 Table — (PDF) [file pgen.1008500.s032.pdf]

S18 Table. Results for meta-analysis of African ancestry cohorts from sequence kernel association test (SKAT) association results for previously reported genes for hemoglobin (HGB), hematocrit (HCT), or white blood cell count (WBC) using TOPMed freeze 5b, Haplotype Reference Consortium (HRC), and 1000G phase 3 as imputation reference panels.

**Gene**	**ENSG ID**	**Chr:Start-End**	**Trait**	**Reference**	**# Variants**	**SKAT P-value**	**N**	**Previous ancestry**	**Previous test type**	**Reference**
HFE	ENSG00000010704	6: 26087281-26098343	HGB	TOPMed freeze 5b	27	2.63E-05	20822	EU+AA	Burden	(17)
				1000G	8	1.96E-05	20822			
				HRC	8	7.25E-05	20822			
MRPL43	ENSG00000055950	10:100969458 - 100987515	HGB	TOPMed freeze 5b	19	0.001	20822	EU	Burden	(17)
				1000G	5	0.013	15244			
				HRC	na	na	na			
MMACHC	ENSG00000132763	1:45500053 - 45513382	HGB	TOPMed freeze 5b	26	0.008	20822	EU	SKAT	(17)
				1000G	5	0.002	20822			
				HRC	na	na	na			
EPO	ENSG00000130427	7: 00720800-100723700	HGB	TOPMed freeze 5b	16	0.005	20822	EA+HA+AA	SKAT	(21)
				1000G	11	0.012	15021			
				HRC	10	0.010	20822			
PKLR	ENSG00000143627	1:155289293-155301434	HGB	TOPMed freeze 5b	27	0.038	20822	Multi	SKAT	(6)
				1000G	5	0.108	1749			
				HRC	10	0.128	20822			
MMACHC	ENSG00000132763	1:45500340-45509214	HCT	TOPMed freeze 5b	26	0.025	20818	EU	SKAT	(17)
				1000G	5	0.008	20818			
				HRC	na	na	na			
PKLR	ENSG00000143627	1:155289293-155301434	HCT	TOPMed freeze 5b	27	0.025	20818	Multi	SKAT	(6)
				1000G	5	0.047	17485			
				HRC	10	0.093	20818			
HFE	ENSG00000010704	6:26087281-26098343	HCT	TOPMed freeze 5b	27	0.001	20818	EU+AA	Burden	(17)
				1000G	8	0.013	20818			
				HRC	8	6.88E-04	20818			
CXCR2	ENSG00000180871	2:218125289-218137253	WBC	TOPMed freeze 5b	21	0.033	20827	EU	Burden	(17)
				1000G	4	0.120	15025			
				HRC	3	0.064	20826			
IQCJ	ENSG00000214216	3:158962235-159897366	WBC	TOPMed freeze 5b	9	0.028	20827	EU+HA+AA	SKAT	(21)
				1000G	2	0.077	20826			
				HRC	na	na	na			
S1PR4	ENSG00000125910	19:3172346-3180332	WBC	TOPMed freeze 5b	33	0.045	20827	EU+HA+AA	SKAT	(21)
				1000G	4	0.137	13659			
				HRC	7	0.066	15025			

JAK2	ENSG00000096968	9:4984390-5128183	WBC	TOPMed freeze 5b	37	0.026	20827	Multi	VT	(19)
				1000G	13	0.008	20826			
				HRC	13	0.042	20826			
TAF3	ENSG00000165632	10:7818504-8016627	WBC	TOPMed freeze 5b	26	0.053	20827	EU	VT	(19)
				1000G	4	0.006	20826			
				HRC	9	0.036	20826			

Previous ancestry column lists the ancestry group in which the significant gene-blood cell trait association signal was previously reported (Multi, multi-ethnic cohort, EU, European, HA, Hispanic/Latino, AA, African American).
Previous test type- statistical test used for previous gene-blood cell trait association (VT, variable threshold, burden, or SKAT).

Association tests were performed using the mmskat function in EPACTS to adjust for kinship, and then p-values were meta-analyzed using metal. Variants were included in an association test if they were annotated as loss of function, high confidence loss of function, missense, protein altering indels, or synonymous variants with fathmm_MKL scores>0.5. Annotation was completed with WGS annotator (WGSA, (22)) using VEP predicted effects for Ensembl(release 83)/GENCODE v24 transcripts and compiled using WGSAParsr version 5.0.4. A variant was included if the minor allele frequency (MAF) was less than 1% in at least one cohort where the estimated r^2 exceeded 0.8, and the MAF was less than 5% in all cohorts where the estimated r^2 exceeded 0.8. A variant was only considered for a particular cohort if the estimated r^2 exceeded 0.8 in that cohort, meaning that the number of variants was allowed to differ by cohort. The number of variants in the table above is the total number of variants included in any SKAT test in the meta-analysis.

All white blood cell results are adjusted for Duffy variant rs2814778. *G6PD* is not included because we only considered autosomal variants in this study.
