# Supplementary material for: Use of >100,000 NHLBI Trans-Omics for Precision Medicine (TOPMed) Consortium whole genome sequences improves imputation quality and detection of rare variant associations in admixed African and Hispanic/Latino populations
Source: PLoS Genet. 2019 Dec 23;15(12):e1008500. doi: 10.1371/journal.pgen.1008500 (PMC6953885; doi:10.1371/journal.pgen.1008500)
Supplement: S19 Table — (PDF) [file pgen.1008500.s033.pdf]

S19 Table. Overall counts for gene results replicated in African ancestry cohorts using TOPMed freeze 5b, 1000G phase 3, and Haplotype Reference Consortium (HRC) as imputation reference panels.

Trait	Imputation Reference	# Genes Tested	# genes with p-value <0.005	# Genes with p-value <0.01	# Genes with p-value <0.05	# Genes with p-value <0.1
**HGB**	TOPMed freeze 5b	5	3	4	5	5
	1000G	5	2	3	4	4
	HRC	3	1	1	2	2
**HCT**	TOPMed freeze 5b	3	1	1	3	3
	1000G	3	0	1	3	3
	HRC	2	1	1	1	2
**WBC**	TOPMed freeze 5b	5	0	0	4	5
	1000G	5	0	2	2	3
	HRC	4	0	0	2	4
**All**	TOPMed freeze 5b	13	4	5	12	13
	1000G	13	2	6	9	10
	HRC	9	2	2	5	8

HCT, hematocrit, HGB, hemoglobin, WBC, white blood cell count
