# Supplementary material for: Use of >100,000 NHLBI Trans-Omics for Precision Medicine (TOPMed) Consortium whole genome sequences improves imputation quality and detection of rare variant associations in admixed African and Hispanic/Latino populations
Source: PLoS Genet. 2019 Dec 23;15(12):e1008500. doi: 10.1371/journal.pgen.1008500 (PMC6953885; doi:10.1371/journal.pgen.1008500)
Supplement: S20 Table — (PDF) [file pgen.1008500.s034.pdf]

S20 Table. Results for meta-analysis of Hispanic/Latino cohorts from sequence kernel association test (SKAT) association results for previously reported genes for hemoglobin (HGB), hematocrit (HCT), or white blood cell count (WBC) using TOPMed freeze 5b, Haplotype Reference Consortium (HRC), and 1000G phase 3 as imputation reference panels.

**Gene**	**ENSG ID**	**Chr:Start-End**	**Trait**	**Reference**	**# Variants**	**SKAT P-value**	**N**	**Previous ancestry**	**Previous test type**	**Reference**
HFE	ENSG00000010704	6: 26087281-26098343	HGB	TOPMed freeze 5b	18	0.085	21644	EU+AA	Burden	(17)
				1000G	7	0.160	21644			
				HRC	8	0.140	21644			
MRPL43	ENSG00000055950	10:100969458 - 100987515	HGB	TOPMed freeze 5b	16	0.299	21644	EU	Burden	(17)
				1000G	10	0.700	14780			
				HRC	na	na	na			
MMACHC	ENSG00000132763	1:45500053 - 45513382	HGB	TOPMed freeze 5b	29	0.323	21644	EU	SKAT	(17)
				1000G	8	0.114	21644			
				HRC	na	na	na			
EPO	ENSG00000130427	7: 00720800-100723700	HGB	TOPMed freeze 5b	12	0.242	21644	EA+HA+AA	SKAT	(21)
				1000G	10	0.164	21644			
				HRC	9	0.042	21644			
PKLR	ENSG00000143627	1:155289293-155301434	HGB	TOPMed freeze 5b	18	0.030	21644	Multi	SKAT	(6)
				1000G	5	0.103	21644			
				HRC	9	0.098	21644			
MMACHC	ENSG00000132763	1:45500340-45509214	HCT	TOPMed freeze 5b	29	0.108	21642	EU	SKAT	(17)
				1000G	8	0.030	21642			
				HRC	na	na	na			
PKLR	ENSG00000143627	1:155289293-155301434	HCT	TOPMed freeze 5b	18	0.097	21642	Multi	SKAT	(6)
				1000G	5	0.327	11546			
				HRC	9	0.345	21642			
HFE	ENSG00000010704	6:26087281-26098343	HCT	TOPMed freeze 5b	18	0.070	21642	EU+AA	Burden	(17)
				1000G	7	0.136	21642			
				HRC	8	0.117	21642			
CXCR2	ENSG00000180871	2:218125289-218137253	WBC	TOPMed freeze 5b	19	0.258	20995	EU	Burden	(17)
				1000G	6	0.349	20995			
				HRC	6	0.276	20995			
IQCJ	ENSG00000214216	3:158962235-159897366	WBC	TOPMed freeze 5b	9	0.272	20995	EA+HA+AA	SKAT	(21)
				1000G	3	0.150	20995			
				HRC	2	0.193	20995			
S1PR4	ENSG00000125910	19:3172346-3180332	WBC	TOPMed freeze 5b	25	0.326	20995	EA+HA+AA	SKAT	(21)
				1000G	3	0.620	14133			

				HRC	7	0.326	20995			
JAK2	ENSG00000096968	9:4984390-5128183	WBC	TOPMed freeze 5b	38	0.011	20995	Multi	VT	(19)
				1000G	17	0.009	20995			
				HRC	22	0.001	20995			
TAF3	ENSG00000165632	10:7818504-8016627	WBC	TOPMed freeze 5b	19	0.034	20995	EU	VT	(19)
				1000G	5	0.076	20995			
				HRC	11	0.068	20995			

Previous ancestry column lists the ancestry group in which the significant gene-blood cell trait association signal was previously reported (Multi, multi-ethnic cohort, EU, European, HA, Hispanic/Latino, AA, African American). Previous test type- statistical test used for previous gene-blood cell trait association (VT, variable threshold, burden, or SKAT).

Association tests were performed using the mmskat function in EPACTS to adjust for kinship, and then p-values were meta-analyzed using metal. Variants were included in an association test if they were annotated as loss of function, high confidence loss of function, missense, protein altering indels, or synonymous variants with fathmm_MKL scores>0.5. Annotation was completed with WGS annotator (WGSA, (22)) using VEP predicted effects for Ensembl (release 83)/GENCODE v24 transcripts and compiled using WGSAParsr version 5.0.4. A variant was included if the minor allele frequency (MAF) was less than 1% in at least one cohort where the estimated r^2 exceeded 0.8, and the MAF was less than 5% in all cohorts where the estimated r^2 exceeded 0.8. A variant was only considered for a particular cohort if the estimated r^2 exceeded 0.8 in that cohort, meaning that the number of variants was allowed to differ by cohort. The number of variants in the table above is the total number of variants included in any SKAT test in the meta-analysis.

All white blood cell results are adjusted for Duffy variant rs2814778. *G6PD* is not included because we only considered autosomal variants in this study.
