# Supplementary material for: Use of >100,000 NHLBI Trans-Omics for Precision Medicine (TOPMed) Consortium whole genome sequences improves imputation quality and detection of rare variant associations in admixed African and Hispanic/Latino populations
Source: PLoS Genet. 2019 Dec 23;15(12):e1008500. doi: 10.1371/journal.pgen.1008500 (PMC6953885; doi:10.1371/journal.pgen.1008500)
Supplement: S21 Table — (PDF) [file pgen.1008500.s035.pdf]

S21 Table. Overall counts for gene results replicated in Hispanic/Latino cohorts using TOPMed freeze 5b, 1000G phase 3, and Haplotype Reference Consortium (HRC) as imputation reference panels.

Trait	Imputation Reference	# Genes Tested	# Genes with p-value <0.005	# Genes with p-value <0.01	# Genes with p-value <0.05	# Genes with p-value <0.1
**HGB**	TOPMed freeze 5b	5	0	0	1	2
	1000G	5	0	0	0	0
	HRC	3	0	0	1	2
**HCT**	TOPMed freeze 5b	3	0	0	0	2
	1000G	3	0	0	1	1
	HRC	2	0	0	0	0
**WBC**	TOPMed freeze 5b	5	0	0	2	2
	1000G	5	0	1	1	2
	HRC	5	0	1	1	2
**All**	TOPMed freeze 5b	13	0	0	3	6
	1000G	13	0	1	2	3
	HRC	9	0	1	2	4

HCT, hematocrit, HGB, hemoglobin, WBC, white blood cell count
