# Supplementary material for: Use of >100,000 NHLBI Trans-Omics for Precision Medicine (TOPMed) Consortium whole genome sequences improves imputation quality and detection of rare variant associations in admixed African and Hispanic/Latino populations
Source: PLoS Genet. 2019 Dec 23;15(12):e1008500. doi: 10.1371/journal.pgen.1008500 (PMC6953885; doi:10.1371/journal.pgen.1008500)
Supplement: S1 File — (PDF) [file pgen.1008500.s036.pdf]

Name	Institution(s)	Primary Department	Institution City	Institution State	Zip Code	Country
Abe, Namiko	New York Genome Center		New York	New York	10013	US
Abecasis, Gonçalo	University of Michigan		Ann Arbor	Michigan	48109	US
Albert, Christine	Massachusetts General Hospital		Boston	Massachusetts	02114	US
Almasy, Laura	Children's Hospital of Philadelphia, University of Pennsylvania		Philadelphia	Pennsylvania	19104	US
Alonso, Alvaro	Emory University		Atlanta	Georgia	30322	US
Ament, Seth	University of Maryland		Baltimore	Maryland	21201	US
Anderson, Peter	University of Washington		Seattle	Washington	98195	US
Anugu, Pramod	University of Mississippi		Jackson	Mississippi	38677	US
Applebaum-Bowden, Deborah	National Institutes of Health		Bethesda	Maryland	20892	US
Arking, Dan	Johns Hopkins University		Baltimore	Maryland	21218	US
Arnett, Donna K	University of Kentucky		Lexington	Kentucky	40506	US
Ashley-Koch, Allison	Duke University		Durham	North Carolina	27708	US

Name	Institution(s)	Primary Department	Institution City	Institution State	Zip Code	Country
Aslibekyan, Stella	University of Alabama		Birmingham	Alabama	35487	US
Assimes, Tim	Stanford University		Stanford	California	94305	US
Auer, Paul	University of Wisconsin Milwaukee		Milwaukee	Wisconsin	53211	US
Avramopoulos, Dimitrios	Johns Hopkins University		Baltimore	Maryland	21218	US
Barnard, John	Cleveland Clinic		Cleveland	Ohio	44195	US
Barnes, Kathleen	University of Colorado at Denver		Denver	Colorado	80204	US
Barr, R. Graham	Columbia University		New York	New York	10027	US
Barron-Casella, Emily	Johns Hopkins University		Baltimore	Maryland	21218	US
Beaty, Terri	Johns Hopkins University		Baltimore	Maryland	21218	US
Becker, Diane	Johns Hopkins University		Baltimore	Maryland	21218	US
Becker, Lewis	Johns Hopkins University		Baltimore	Maryland	21218	US
Beer, Rebecca	National Heart, Lung, and Blood Institute, National Institutes of Health		Bethesda	Maryland	20892	US

Name	Institution(s)	Primary Department	Institution City	Institution State	Zip Code	Country
Begum, Ferdouse	Johns Hopkins University		Baltimore	Maryland	21218	US
Beitelshees, Amber	University of Maryland		Baltimore	Maryland	21201	US
Benjamin, Emelia	Boston University, Massachusetts General Hospital	Boston University School of Medicine	Boston	Massachusetts	02118	US
Bezerra, Marcos	Fundação de Hematologia e Hemoterapia de Pernambuco - Hemope		Recife		52011-000	BR
Bielak, Larry	University of Michigan		Ann Arbor	Michigan	48109	US
Bis, Joshua	University of Washington		Seattle	Washington	98195	US
Blackwell, Thomas	University of Michigan		Ann Arbor	Michigan	48109	US
Blangero, John	University of Texas Rio Grande Valley School of Medicine	Human Genetics	Brownsville	Texas	78520	US
Boerwinkle, Eric	University of Texas Health at Houston		Houston	Texas	77225	US
Bowden, Donald W.	Wake Forest Baptist Health	Department of Biochemistry	Winston-Salem	North Carolina	27157	US
Bowler, Russell	National Jewish Health	National Jewish Health	Denver	Colorado	80206	US
Brody, Jennifer	University of Washington		Seattle	Washington	98195	US

Name	Institution(s)	Primary Department	Institution City	Institution State	Zip Code	Country
Broeckel, Ulrich	Medical College of Wisconsin		Milwaukee	Wisconsin	53226	US
Broome, Jai	University of Washington		Seattle	Washington	98195	US
Bunting, Karen	New York Genome Center		New York	New York	10013	US
Burchard, Esteban	University of California, San Francisco		San Francisco	California	94143	US
Buth, Erin	University of Washington	Biostatistics	Seattle	Washington	98195	US
Cade, Brian	Brigham & Women's Hospital	Brigham and Women's Hospital	Boston	Massachusetts	02115	US
Cardwell, Jonathan	University of Colorado at Denver		Denver	Colorado	80204	US
Carty, Cara	Women's Health Initiative		Seattle	Washington	98109	US
Casaburi, Richard	University of California, Los Angeles		Los Angeles	California	90095	US
Casella, James	Johns Hopkins University		Baltimore	Maryland	21218	US
Chaffin, Mark	Broad Institute		Cambridge	Massachusetts	02142	US
Chang, Christy	University of Maryland		Baltimore	Maryland	21201	US
Chasman, Daniel	Brigham & Women's Hospital		Boston	Massachusetts	02115	US

Name	Institution(s)	Primary Department	Institution City	Institution State	Zip Code	Country
Chavan, Sameer	University of Colorado at Denver		Denver	Colorado	80204	US
Chen, Bo-Juen	New York Genome Center		New York	New York	10013	US
Chen, Wei-Min	University of Virginia		Charlottesville	Virginia	22903	US
Chen, Yii-Der Ida	Los Angeles Biomedical Research Institute		Charlottesville	Virginia	90502	US
Cho, Michael	Brigham & Women's Hospital		Boston	Massachusetts	02115	US
Choi, Seung Hoan	Broad Institute		Cambridge	Massachusetts	02142	US
Chuang, Lee-Ming	National Taiwan University	National Taiwan University Hospital	Taipei		10617	TW
Chung, Mina	Cleveland Clinic		Cleveland	Ohio	44195	US
Conomos, Matthew	University of Washington	Biostatistics	Seattle	Washington	98115	US
Cornell, Elaine	University of Vermont		Burlington	Vermont	05405	US
Correa, Adolfo	University of Mississippi	Medicine	Jackson	Mississippi	39216	US
Crandall, Carolyn	University of California, Los Angeles		Los Angeles	California	90095	US
Crapo, James	National Jewish Health		Denver	Colorado	80206	US

Name	Institution(s)	Primary Department	Institution City	Institution State	Zip Code	Country
Cupples, L. Adrienne	Boston University		Boston	Massachusetts	02215	US
Curran, Joanne	University of Texas Rio Grande Valley School of Medicine		Brownsville	Texas	78520	US
Curtis, Jeffrey	University of Michigan		Ann Arbor	Michigan	48109	US
Custer, Brian	Vitalant Research Institute		San Francisco	California	94118	US
Damcott, Coleen	University of Maryland		Baltimore	Maryland	21201	US
Darbar, Dawood	University of Illinois at Chicago		Chicago	Illinois	60607	US
Das, Sayantan	University of Michigan		Ann Arbor	Michigan	48109	US
David, Sean	Stanford University		Stanford	California	94305	US
Davis, Colleen	University of Washington		Seattle	Washington	98195	US
Daya, Michelle	University of Colorado at Denver		Denver	Colorado	80204	US
de Andrade, Mariza	Mayo Clinic		Rochester	Minnesota	55905	US
DeBaun, Michael	Vanderbilt University		Nashville	Tennessee	37235	US
Deka, Ranjan	University of Cincinnati		Cincinnati	Ohio	45220	US

Name	Institution(s)	Primary Department	Institution City	Institution State	Zip Code	Country
DeMeo, Dawn	Brigham & Women's Hospital		Boston	Massachusetts	02115	US
Devine, Scott	University of Maryland		Baltimore	Maryland	21201	US
Do, Ron	Icahn School of Medicine at Mount Sinai		New York	New York	10029	US
Duan, Qing	University of North Carolina		Chapel Hill	North Carolina	27599	US
Duggirala, Ravi	University of Texas Rio Grande Valley School of Medicine		Edinburg	Texas	78539	US
Durda, Jon Peter	University of Vermont		Burlington	Vermont	05405	US
Dutcher, Susan	Washington University in St Louis		St Louis	Missouri	63130	US
Eaton, Charles	Brown University		Providence	Rhode Island	02912	US
Ekunwe, Lynette	University of Mississippi		Jackson	Mississippi	38677	US
Ellinor, Patrick	Massachusetts General Hospital		Boston	Massachusetts	02114	US
Emery, Leslie	University of Washington		Seattle	Washington	98195	US
Farber, Charles	University of Virginia		Charlottesville	Virginia	22903	US
Farnam, Leanna	Brigham & Women's Hospital		Boston	Massachusetts	02115	US

Name	Institution(s)	Primary Department	Institution City	Institution State	Zip Code	Country
Fingerlin, Tasha	National Jewish Health	Center for Genes, Environment and Health	Denver	Colorado	80206	US
Flickinger, Matthew	University of Michigan		Ann Arbor	Michigan	48109	US
Fornage, Myriam	University of Texas Health at Houston		Houston	Texas	77225	US
Franceschini, Nora	University of North Carolina		Chapel Hill	North Carolina	27599	US
Fu, Mao	University of Maryland		Baltimore	Maryland	21201	US
Fullerton, Stephanie M.	University of Washington		Seattle	Washington	98195	US
Fulton, Lucinda	Washington University in St Louis		St Louis	Missouri	63130	US
Gabriel, Stacey	Broad Institute		Cambridge	Massachusetts	02142	US
Gan, Weiniu	National Heart, Lung, and Blood Institute, National Institutes of Health		Bethesda	Maryland	20892	US
Gao, Yan	University of Mississippi		Jackson	Mississippi	38677	US
Gass, Margery	Fred Hutchinson Cancer Research Center		Seattle	Washington	98109	US

Name	Institution(s)	Primary Department	Institution City	Institution State	Zip Code	Country
Gelb, Bruce	Icahn School of Medicine at Mount Sinai		New York	New York	10029	US
Geng, Xiaoqi (Priscilla)	University of Michigan		Ann Arbor	Michigan	48109	US
Germer, Soren	New York Genome Center		New York	New York	10013	US
Gignoux, Chris	Stanford University		Stanford	California	94305	US
Gladwin, Mark	University of Pittsburgh		Pittsburgh	Pennsylvania	15260	US
Glahn, David	Yale University		New Haven	Connecticut	06520	US
Gogarten, Stephanie	University of Washington		Seattle	Washington	98195	US
Gong, Da-Wei	University of Maryland		Baltimore	Maryland	21201	US
Goring, Harald	University of Texas Rio Grande Valley School of Medicine		San Antonio	Texas	78229	US
Gu, C. Charles	Washington University in St Louis		St Louis	Missouri	63130	US
Guan, Yue	University of Maryland		Baltimore	Maryland	21201	US
Guo, Xiuqing	Los Angeles Biomedical Research Institute		Los Angeles	California	90502	US

Name	Institution(s)	Primary Department	Institution City	Institution State	Zip Code	Country
Haessler, Jeff	Fred Hutchinson Cancer Research Center, Women's Health Initiative		Seattle	Washington	98109	US
Hall, Michael	University of Mississippi		Jackson	Mississippi	38677	US
Harris, Daniel	University of Maryland		Baltimore	Maryland	21201	US
Hawley, Nicola	Yale University		New Haven	Connecticut	06520	US
He, Jiang	Tulane University		New Orleans	Louisiana	70118	US
Heavner, Ben	University of Washington	Biostatistics	Seattle	Washington	98195	US
Heckbert, Susan	University of Washington		Seattle	Washington	98195	US
Hernandez, Ryan	McGill University, University of California, San Francisco					CA
Herrington, David	Wake Forest Baptist Health		Winston-Salem	North Carolina	27157	US
Hersh, Craig	Brigham & Women's Hospital	Channing Division of Network Medicine	Boston	Massachusetts	02115	US
Hidalgo, Bertha	University of Alabama		Birmingham	Alabama	35487	US
Hixson, James	University of Texas Health at Houston		Houston	Texas	77225	US

Name	Institution(s)	Primary Department	Institution City	Institution State	Zip Code	Country
Hokanson, John	University of Colorado at Denver		Denver	Colorado	80204	US
Hong, Elliott	University of Maryland		Baltimore	Maryland	21201	US
Hoth, Karin	University of Iowa		Iowa City	Iowa	52242	US
Hsiung, Chao (Agnes)	National Health Research Institute Taiwan	Institute of Population Health Sciences, NHRI	Miaoli County		350	TW
Huston, Haley	Blood Works Northwest		Seattle	Washington	98105	US
Hwu, Chii Min	Taichung Veterans General Hospital Taiwan		Taichung City		407	TW
Irvin, Marguerite Ryan	University of Alabama		Birmingham	Alabama	35487	US
Jackson, Rebecca	Ohio State University Wexner Medical Center	Internal Medicine, DIvision of Endocrinology, Diabetes and Metabolism	Columbus	Ohio	43210	US
Jain, Deepti	University of Washington		Seattle	Washington	98195	US
Jaquish, Cashell	National Heart, Lung, and Blood Institute, National Institutes of Health		Bethesda	Maryland	20892	US
Jhun, Min A	University of Michigan		Ann Arbor	Michigan	48109	US

Name	Institution(s)	Primary Department	Institution City	Institution State	Zip Code	Country
Johnsen, Jill	Blood Works Northwest, University of Washington		Seattle	Washington	98106	US
Johnson, Andrew	National Heart, Lung, and Blood Institute, National Institutes of Health		Bethesda	Maryland	20892	US
Johnson, Craig	University of Washington		Seattle	Washington	98195	US
Johnston, Rich	Emory University		Atlanta	Georgia	30322	US
Jones, Kimberly	Johns Hopkins University		Baltimore	Maryland	21218	US
Kang, Hyun Min	University of Michigan	Biostatistics	Ann Arbor	Michigan	48109	US
Kaplan, Robert	Albert Einstein College of Medicine		New York	New York	10461	US
Kardia, Sharon	University of Michigan		Ann Arbor	Michigan	48109	US
Kathiresan, Sekar	Broad Institute		Cambridge	Massachusetts	02142	US
Kaufman, Laura	Brigham & Women's Hospital		Boston	Massachusetts	02115	US
Kelly, Shannon	Vitalant Research Institute		San Francisco	California	94118	US
Kenny, Eimear	Icahn School of Medicine at Mount Sinai		New York	New York	10029	US

Name	Institution(s)	Primary Department	Institution City	Institution State	Zip Code	Country
Kessler, Michael	University of Maryland		Baltimore	Maryland	21201	US
Khan, Alyna	University of Washington		Seattle	Washington	98195	US
Kinney, Greg	University of Colorado at Denver		Denver	Colorado	80204	US
Konkle, Barbara	Blood Works Northwest		Seattle	Washington	98104	US
Kooperberg, Charles	Fred Hutchinson Cancer Research Center		Seattle	Washington	98109	US
Kramer, Holly	Loyola University	Public Health Sciences	Maywood	Illinois	60153	US
Krauter, Stephanie	University of Washington		Seattle	Washington	98195	US
Lange, Christoph	Harvard School of Public Health	Biostats	Boston	Massachusetts	02115	US
Lange, Ethan	University of Colorado at Denver		Denver	Colorado	80204	US
Lange, Leslie	University of Colorado at Denver		Denver	Colorado	80204	US
Laurie, Cathy	University of Washington		Seattle	Washington	98195	US
Laurie, Cecelia	University of Washington		Seattle	Washington	98195	US

Name	Institution(s)	Primary Department	Institution City	Institution State	Zip Code	Country
LeBoff, Meryl	Brigham & Women's Hospital		Boston	Massachusetts	02115	US
Lee, Jiwon	Brigham & Women's Hospital					
Lee, Seunggeun Shawn	University of Michigan		Ann Arbor	Michigan	48109	US
Lee, Wen-Jane	Taichung Veterans General Hospital Taiwan		Taichung City		407	TW
LeFaive, Jonathon	University of Michigan		Ann Arbor	Michigan	48109	US
Levine, David	University of Washington		Seattle	Washington	98195	US
Levy, Dan	National Heart, Lung, and Blood Institute, National Institutes of Health		Bethesda	Maryland	20892	US
Lewis, Joshua	University of Maryland		Baltimore	Maryland	21201	US
Li, Yun	University of North Carolina		Chapel Hill	North Carolina	27599	US
Lin, Honghuang	Boston University		Boston	Massachusetts	02215	US
Lin, Keng Han	University of Michigan		Ann Arbor	Michigan	48109	US
Lin, Xihong	Harvard School of Public Health					

Name	Institution(s)	Primary Department	Institution City	Institution State	Zip Code	Country
Liu, Simin	Brown University, Women's Health Initiative	Epidemiology	Providence	Rhode Island	02912	US
Liu, Yongmei	Wake Forest Baptist Health		Winston-Salem	North Carolina	27157	US
Loos, Ruth	Icahn School of Medicine at Mount Sinai		New York	New York	10029	US
Lubitz, Steven	Massachusetts General Hospital		Boston	Massachusetts	02114	US
Lunetta, Kathryn	Boston University		Boston	Massachusetts	02215	US
Luo, James	National Heart, Lung, and Blood Institute, National Institutes of Health		Bethesda	Maryland	20892	US
Mahaney, Michael	University of Texas Rio Grande Valley School of Medicine		Brownsville	Texas	78520	US
Make, Barry	Johns Hopkins University		Baltimore	Maryland	21218	US
Manichaikul, Ani	University of Virginia		Charlottesville	Virginia	22903	US
Manson, JoAnn	Brigham & Women's Hospital		Boston	Massachusetts	02115	US
Margolin, Lauren	Broad Institute		Cambridge	Massachusetts	02142	US

Name	Institution(s)	Primary Department	Institution City	Institution State	Zip Code	Country
Martin, Lisa	George Washington University		Washington	District of Columbia	20052	US
Mathai, Susan	University of Colorado at Denver		Denver	Colorado	80204	US
Mathias, Rasika	Johns Hopkins University		Baltimore	Maryland	21218	US
McArdle, Patrick	University of Maryland		Baltimore	Maryland	21201	US
McDonald, Merry-Lynn	University of Alabama		Birmingham	Alabama	35487	US
McFarland, Sean	Harvard University		Cambridge	Massachusetts	02138	US
McGarvey, Stephen	Brown University		Providence	Rhode Island	02912	US
McHugh, Caitlin	University of Washington	Biostatistics	Seattle	Washington	98145	US
Mei, Hao	University of Mississippi		Jackson	Mississippi	38677	US
Meyers, Deborah A	University of Arizona		Tucson	Arizona	85721	US
Mikulla, Julie	National Heart, Lung, and Blood Institute, National Institutes of Health		Bethesda	Maryland	20892	US
Min, Nancy	University of Mississippi		Jackson	Mississippi	38677	US

Name	Institution(s)	Primary Department	Institution City	Institution State	Zip Code	Country
Minear, Mollie	National Heart, Lung, and Blood Institute, National Institutes of Health		Bethesda	Maryland	20892	US
Minster, Ryan L	University of Pittsburgh		Pittsburgh	Pennsylvania	15260	US
Mitchell, Braxton D.	University of Maryland		Baltimore	Maryland	21201	US
Montasser, May E.	University of Maryland		Baltimore	Maryland	21201	US
Musani, Solomon	University of Mississippi	Medicine	Jackson	Mississippi	39213	US
Mwasongwe, Stanford	University of Mississippi		Jackson	Mississippi	38677	US
Mychaleckyj, Josyf C	University of Virginia		Charlottesville	Virginia	22903	US
Nadkarni, Girish	Icahn School of Medicine at Mount Sinai		New York	New York	10029	US
Naik, Rakhi	Johns Hopkins University		Baltimore	Maryland	21218	US
Naseri, Take	Ministry of Health, Government of Samoa		Apia			WS

Name	Institution(s)	Primary Department	Institution City	Institution State	Zip Code	Country
Natarajan, Pradeep	Broad Institute, Harvard University, Massachusetts General Hospital		Cambridge	Massachusetts	02138	US
Nekhai, Sergei	Howard University		Washington	District of Columbia	20059	US
Nelson, Sarah C.	University of Washington	Biostatistics	Seattle	Washington	98195	US
Nickerson, Deborah	University of Washington		Seattle	Washington	98195	US
North, Kari	University of North Carolina		Chapel Hill	North Carolina	27599	US
O'Connell, Jeff	University of Maryland		Balitmore	Maryland	21201	US
O'Connor, Tim	University of Maryland		Baltimore	Maryland	21201	US
Ochs-Balcom, Heather	University at Buffalo		Buffalo	New York	14260	US
Palmer, Nicholette	Wake Forest Baptist Health	Biochemistry	Winston-Salem	North Carolina	27157	US
Pankow, James	University of Minnesota		Minneapolis	Minnesota	55455	US
Papanicolaou, George	National Heart, Lung, and Blood Institute, National Institutes of Health		Bethesda	Maryland	20892	US

Name	Institution(s)	Primary Department	Institution City	Institution State	Zip Code	Country
Parker, Margaret	Brigham & Women's Hospital		Boston	Massachusetts	02115	US
Parsa, Afshin	University of Maryland		Baltimore	Maryland	21201	US
Penchev, Sara	National Jewish Health		Denver	Colorado	80206	US
Peralta, Juan Manuel	University of Texas Rio Grande Valley School of Medicine		Edinburg	Texas	78539	US
Perez, Marco	Stanford University		Stanford	California	94305	US
Perry, James	University of Maryland		Baltimore	Maryland	21201	US
Peters, Ulrike	Fred Hutchinson Cancer Research Center, University of Washington		Seattle	Washington	98109	US
Peyser, Patricia	University of Michigan		Ann Arbor	Michigan	48109	US
Phillips, Lawrence S	Emory University		Atlanta	Georgia	30322	US
Phillips, Sam	University of Washington		Seattle	Washington	98195	US
Pollin, Toni	University of Maryland		Baltimore	Maryland	21201	US
Post, Wendy	Johns Hopkins University	Cardiology/Medicine	Baltimore	Maryland	21218	US

Name	Institution(s)	Primary Department	Institution City	Institution State	Zip Code	Country
Powers Becker, Julia	University of Colorado at Denver	Medicine	Denver	Colorado	80204	US
Preethi Boorgula, Meher	University of Colorado at Denver		Denver	Colorado	80204	US
Preuss, Michael	Icahn School of Medicine at Mount Sinai		New York	New York	10029	US
Prokopenko, Dmitry	Harvard University		Cambridge	Massachusetts	02138	US
Psaty, Bruce	University of Washington		Seattle	Washington	98195	US
Qasba, Pankaj	National Heart, Lung, and Blood Institute, National Institutes of Health		Bethesda	Maryland	20892	US
Qiao, Dandi	Brigham & Women's Hospital		Boston	Massachusetts	02115	US
Qin, Zhaohui	Emory University		Atlanta	Georgia	30322	US
Rafaels, Nicholas	University of Colorado at Denver		Denver	Colorado	80045	US
Raffield, Laura	University of North Carolina	Genetics	Chapel Hill	North Carolina	27599	US
Rao, D.C.	Washington University in St Louis		St Louis	Missouri	63130	US

Name	Institution(s)	Primary Department	Institution City	Institution State	Zip Code	Country
Rasmussen-Torvik, Laura	Northwestern University		Chicago	Illinois	60208	US
Ratan, Aakrosh	University of Virginia		Charlottesville	Virginia	22903	US
Redline, Susan	Brigham & Women's Hospital		Boston	Massachusetts	02115	US
Reed, Robert	University of Maryland		Baltimore	Maryland	21201	US
Regan, Elizabeth	National Jewish Health		Denver	Colorado	80206	US
Reiner, Alex	Fred Hutchinson Cancer Research Center, University of Washington		Seattle	Washington	98109	US
Reupena, Muagututi‘a Sefuiva	Lutia I Puava Ae Mapu I Fagalele		Apia			WS
Rice, Ken	University of Washington		Seattle	Washington	98195	US
Rich, Stephen	University of Virginia		Charlottesville	Virginia	22903	US
Roden, Dan	Vanderbilt University	Medicine, Pharmacology, Biomedicla Informatics	Nashville	Tennessee	37235	US
Roselli, Carolina	Broad Institute		Cambridge	Massachusetts	02142	US
Rotter, Jerome	Los Angeles Biomedical Research Institute		Los Angeles	California	90502	US

Name	Institution(s)	Primary Department	Institution City	Institution State	Zip Code	Country
Ruczinski, Ingo	Johns Hopkins University		Baltimore	Maryland	21218	US
Russell, Pamela	University of Colorado at Denver		Denver	Colorado	80204	US
Ruuska, Sarah	Blood Works Northwest		Seattle	Washington	98107	US
Ryan, Kathleen	University of Maryland		Baltimore	Maryland	21201	US
Sabino, Ester Cerdeira	Universidade de Sao Paulo	Faculdade de Medicina	Sao Paulo		01310000	BR
Sakornsakolpat, Phuwanat	Brigham & Women's Hospital		Boston	Massachusetts	02115	US
Salimi, Shabnam	University of Maryland		Baltimore	Maryland	21201	US
Salzberg, Steven	Johns Hopkins University		Baltimore	Maryland	21218	US
Sandow, Kevin	Los Angeles Biomedical Research Institute	TGPS	Torrance	California	90502	US
Sankaran, Vijay	Harvard University		Cambridge	Massachusetts	02138	US
Scheller, Christopher	University of Michigan		Ann Arbor	Michigan	48109	US
Schmidt, Ellen	University of Michigan		Ann Arbor	Michigan	48109	US

Name	Institution(s)	Primary Department	Institution City	Institution State	Zip Code	Country
Schwander, Karen	Washington University in St Louis		St Louis	Missouri	63130	US
Schwartz, David	University of Colorado at Denver		Denver	Colorado	80204	US
Sciurba, Frank	University of Pittsburgh		Pittsburgh	Pennsylvania	15260	US
Seidman, Christine	Harvard Medical School	Genetics	Boston	Massachusetts	02115	US
Seidman, Jonathan	Harvard Medical School					
Sheehan, Vivien	Baylor College of Medicine	Pediatrics	Houston	Texas	77030	US
Shetty, Amol	University of Maryland		Baltimore	Maryland	21201	US
Shetty, Aniket	University of Colorado at Denver		Denver	Colorado	80204	US
Sheu, Wayne Hui-Heng	Taichung Veterans General Hospital Taiwan		Taichung City		407	TW
Shoemaker, M. Benjamin	Vanderbilt University		Nashville	Tennessee	37235	US
Silver, Brian	UMass Memorial Medical Center		Worcester	Massachusetts	01655	US
Silverman, Edwin	Brigham & Women's Hospital		Boston	Massachusetts	02115	US

Name	Institution(s)	Primary Department	Institution City	Institution State	Zip Code	Country
Smith, Jennifer	University of Michigan		Ann Arbor	Michigan	48109	US
Smith, Josh	University of Washington		Seattle	Washington	98195	US
Smith, Nicholas	University of Washington		Seattle	Washington	98195	US
Smith, Tanja	New York Genome Center		New York	New York	10013	US
Smoller, Sylvia	Albert Einstein College of Medicine		New York	New York	10461	US
Snively, Beverly	Wake Forest Baptist Health	Biostatistical Sciences	Winston-Salem	North Carolina	27157	US
Sofer, Tamar	Brigham & Women's Hospital		Boston	Massachusetts	02115	US
Sotoodehnia, Nona	University of Washington		Seattle	Washington	98195	US
Stilp, Adrienne	University of Washington		Seattle	Washington	98195	US
Streeten, Elizabeth	University of Maryland		Baltimore	Maryland	21201	US
Su, Jessica Lasky	Brigham & Women's Hospital					
Sung, Yun Ju	Washington University in St Louis		St Louis	Missouri	63130	US
Sylvia, Jody	Brigham & Women's Hospital		Boston	Massachusetts	02115	US

Name	Institution(s)	Primary Department	Institution City	Institution State	Zip Code	Country
Szpiro, Adam	University of Washington		Seattle	Washington	98195	US
Sztalryd, Carole	University of Maryland		Baltimore	Maryland	21201	US
Taliun, Daniel	University of Michigan		Ann Arbor	Michigan	48109	US
Tang, Hua	Stanford University	Genetics	Stanford	California	94305	US
Taub, Margaret	Johns Hopkins University		Baltimore	Maryland	21218	US
Taylor, Kent D.	Los Angeles Biomedical Research Institute	Institute for Translational Genomics and Populations Sciences	Torrance	California	90502	US
Taylor, Simeon	University of Maryland		Baltimore	Maryland	21201	US
Telen, Marilyn	Duke University		Durham	North Carolina	27708	US
Thornton, Timothy A.	University of Washington		Seattle	Washington	98195	US
Tinker, Lesley	Women's Health Initiative		Seattle	Washington	98109	US
Tirschwell, David	University of Washington		Seattle	Washington	98195	US
Tiwari, Hemant	University of Alabama		Birmingham	Alabama	35487	US
Tracy, Russell	University of Vermont	Pathology & Laboratory Medicine	Burlington	Vermont	05405	US
Tsai, Michael	University of Minnesota		Minneapolis	Minnesota	55455	US

Name	Institution(s)	Primary Department	Institution City	Institution State	Zip Code	Country
Vaidya, Dhananjay	Johns Hopkins University		Baltimore	Maryland	21218	US
VandeHaar, Peter	University of Michigan		Ann Arbor	Michigan	48109	US
Vasan, Ramachandran S.	Boston University		Boston	Massachusetts	02215	US
Vrieze, Scott	University of Colorado at Boulder, University of Minnesota		Boulder	Colorado	80309	US
Walker, Tarik	University of Colorado at Denver		Denver	Colorado	80204	US
Wallace, Robert	University of Iowa		Iowa City	Iowa	52242	US
Walts, Avram	University of Colorado at Denver		Denver	Colorado	80204	US
Wan, Emily	Brigham & Women's Hospital		Boston	Massachusetts	02115	US
Wang, Fei Fei	University of Washington		Seattle	Washington	98195	US
Wang, Heming	Brigham & Women's Hospital, Partners.org					
Watson, Karol	University of California, Los Angeles		Los Angeles	California	90095	US

Name	Institution(s)	Primary Department	Institution City	Institution State	Zip Code	Country
Weeks, Daniel E.	University of Pittsburgh		Pittsburgh	Pennsylvania	15260	US
Weir, Bruce	University of Washington		Seattle	Washington	98195	US
Weiss, Scott	Brigham & Women's Hospital		Boston	Massachusetts	02115	US
Weng, Lu-Chen	Massachusetts General Hospital		Boston	Massachusetts	02114	US
Willer, Cristen	University of Michigan	Internal Medicine	Ann Arbor	Michigan	48109	US
Williams, Kayleen	University of Washington		Seattle	Washington	98195	US
Williams, L. Keoki	Henry Ford Health System		Detroit	Michigan	48202	US
Wilson, Carla	Brigham & Women's Hospital		Boston	Massachusetts	02115	US
Wilson, James	University of Mississippi	Department of Physiology and Biophysics	Jackson	Mississippi	39216	US
Wong, Quenna	University of Washington		Seattle	Washington	98195	US
Xu, Huichun	University of Maryland		Baltimore	Maryland	21201	US
Yanek, Lisa	Johns Hopkins University		Baltimore	Maryland	21218	US
Yang, Ivana	University of Colorado at Denver		Denver	Colorado	80204	US
Yang, Rongze	University of Maryland		Baltimore	Maryland	21201	US

Name	Institution(s)	Primary Department	Institution City	Institution State	Zip Code	Country
Zaghloul, Norann	University of Maryland		Baltimore	Maryland	21201	US
Zekavat, Maryam	Broad Institute		Cambridge	Massachusetts	02142	US
Zhang, Yingze	University of Pittsburgh	Medicine	Pittsburgh	Pennsylvania	15260	US
Zhao, Snow Xueyan	National Jewish Health		Denver	Colorado	80206	US
Zhao, Wei	University of Michigan		Ann Arbor	Michigan	48109	US
Zhi, Degui	University of Texas Health at Houston		Houston	Texas	77225	US
Zhou, Xiang	University of Michigan		Ann Arbor	Michigan	48109	US
Zhu, Xiaofeng	Case Western Reserve University	Department of Population and Quantitative Health Sciences	Cleveland	Ohio	44106	US
Zody, Michael	New York Genome Center		New York	New York	10013	US
Zoellner, Sebastian	University of Michigan		Ann Arbor	Michigan	48109	US
