# Supplementary material for: Use of >100,000 NHLBI Trans-Omics for Precision Medicine (TOPMed) Consortium whole genome sequences improves imputation quality and detection of rare variant associations in admixed African and Hispanic/Latino populations
Source: PLoS Genet. 2019 Dec 23;15(12):e1008500. doi: 10.1371/journal.pgen.1008500 (PMC6953885; doi:10.1371/journal.pgen.1008500)
Supplement: S2 File — (PDF) [file pgen.1008500.s037.pdf]

Laura Almasy

Allison Ashley-Koch

Paul Auer

Abraham Aviv

Emily Barron-Casella

David Beame

Lewis Becker

Alexander Bick

Larry Bielak

Thomas Blackwell

John Blangero

Michael Bowers

Jennifer Brody

Jai Broome

James Casella

Christy Chang

Ming-Huei Chen

Han Chen

Michael Cho

Matthew Conomos

Adolfo Correa

Paul de Vries

Pinkal Desai

Qing Duan

Nauder Faraday

Jean Feng

Annette Fitzpatrick

James Floyd

Santhi Ganesh

Brady Gaynor

Manjit Hanspal

Ross Hardison

Ben Heavner

Craig Hersh

Chani Hodonsky

Steve Horvath

Yao Hu

Jennifer Huffman

Kruthika Raman Iyer

Deepti Jain

Sidd Jaiswal

Jill Johnsen

Andrew Johnson

Brian Joyce

Shannon Kelly

Derek Klarin

Malgorzata Klauzinska

Barbara Konkle

Charles Kooperberg

Ethan Lange

Leslie Lange

Cecelia Laurie

Cathy Laurie

Grace Lee

Maarten Leerkes

Guillaume Lettre

David Levine

Dan Levy

Joshua Lewis

Yun Li

Bingshan Li

Amarise Little

Rasika Mathias

Caitlin McHugh

Karen Miga

Anna Mikhaylova

Julie Mikulla

Braxton D. Mitchell

Alanna C Morrison

Rakhi Naik

Drew Nannini

Pradeep Natarajan

Deborah Nickerson

Jeff O'Connell

Christopher O'Donnell

Nels Olson

Nathan Pankratz

Benedict Paten

James Perry

Steve Pipe

Linda Polfus

Bruce Psaty

Jenn Purnell

Laura Raffield

Alex Reiner

Stephen Rich

Benjamin Andrew Thomas Rodriguez

Shabnam Salimi

Vijay Sankaran

Noah Simon

Nicholas Smith

Adrienne Stilp

Hua Tang

Margaret Taub

Marilyn Telen

Timothy A. Thornton

Russell Tracy

Kate Wehr

Joshua Weinstock

Ellen Werner

Marsha Wheeler

Kerri Wiggins

Lisa Yanek

Yu-Chung Yang

Maryam Zekavat

Wei Zhao

Xiuwen Zheng

Yinan Zheng
